# Phased Whole-Genome Genetic Risk in a Family Quartet Using a Major Allele Reference Sequence

**DOI:** 10.1371/journal.pgen.1002280

**Published:** 2011-09-15

**Authors:** Frederick E. Dewey, Rong Chen, Sergio P. Cordero, Kelly E. Ormond, Colleen Caleshu, Konrad J. Karczewski, Michelle Whirl-Carrillo, Matthew T. Wheeler, Joel T. Dudley, Jake K. Byrnes, Omar E. Cornejo, Joshua W. Knowles, Mark Woon, Katrin Sangkuhl, Li Gong, Caroline F. Thorn, Joan M. Hebert, Emidio Capriotti, Sean P. David, Aleksandra Pavlovic, Anne West, Joseph V. Thakuria, Madeleine P. Ball, Alexander W. Zaranek, Heidi L. Rehm, George M. Church, John S. West, Carlos D. Bustamante, Michael Snyder, Russ B. Altman, Teri E. Klein, Atul J. Butte, Euan A. Ashley

**Affiliations:** 1 Center for Inherited Cardiovascular Disease, Division of Cardiovascular Medicine, Stanford University, Stanford, California, United States of America; 2 Division of Systems Medicine, Department of Pediatrics, Stanford University School of Medicine, Stanford, California, United States of America; 3 Biomedical Informatics Graduate Training Program, Stanford University School of Medicine, Stanford, California, United States of America; 4 Department of Genetics, Stanford University School of Medicine, Stanford, California, United States of America; 5 Center for Biomedical Ethics, Stanford University, Stanford, California, United States of America; 6 Wellesley College, Wellesley, Massachusetts, United States of America; 7 Division of Genetics, Massachusetts General Hospital, Boston, Massachusetts, United States of America; 8 Department of Genetics, Harvard Medical School, Boston, Massachusetts, United States of America; 9 Department of Pathology, Harvard Medical School, Boston, Massachusetts, United States of America; 10 Personalis, Palo Alto, California, United States of America; 11 Department of Bioengineering, Stanford University, Stanford, California, United States of America; The University of North Carolina at Chapel Hill, United States of America

## Abstract

Whole-genome sequencing harbors unprecedented potential for characterization of individual and family genetic variation. Here, we develop a novel synthetic human reference sequence that is ethnically concordant and use it for the analysis of genomes from a nuclear family with history of familial thrombophilia. We demonstrate that the use of the major allele reference sequence results in improved genotype accuracy for disease-associated variant loci. We infer recombination sites to the lowest median resolution demonstrated to date (<1,000 base pairs). We use family inheritance state analysis to control sequencing error and inform family-wide haplotype phasing, allowing quantification of genome-wide compound heterozygosity. We develop a sequence-based methodology for Human Leukocyte Antigen typing that contributes to disease risk prediction. Finally, we advance methods for analysis of disease and pharmacogenomic risk across the coding and non-coding genome that incorporate phased variant data. We show these methods are capable of identifying multigenic risk for inherited thrombophilia and informing the appropriate pharmacological therapy. These ethnicity-specific, family-based approaches to interpretation of genetic variation are emblematic of the next generation of genetic risk assessment using whole-genome sequencing.

## Author Summary

An individual's genetic profile plays an important role in determining risk for disease and response to medical therapy. The development of technologies that facilitate rapid whole-genome sequencing will provide unprecedented power in the estimation of disease risk. Here we develop methods to characterize genetic determinants of disease risk and response to medical therapy in a nuclear family of four, leveraging population genetic profiles from recent large scale sequencing projects. We identify the way in which genetic information flows through the family to identify sequencing errors and inheritance patterns of genes contributing to disease risk. In doing so we identify genetic risk factors associated with an inherited predisposition to blood clot formation and response to blood thinning medications. We find that this aligns precisely with the most significant disease to occur to date in the family, namely pulmonary embolism, a blood clot in the lung. These ethnicity-specific, family-based approaches to interpretation of individual genetic profiles are emblematic of the next generation of genetic risk assessment using whole-genome sequencing.

## Introduction

Whole genome sequencing of related individuals provides a window into human recombination as well as superior error control and the ability to phase genomes assembled from high throughput short read sequencing technologies. The interrogation of the entire euchromatic genome, as opposed to the coding exome, provides superior sensitivity to recombination events, allows for full interrogation of regulatory regions, and comprehensive exploration of disease associated variant loci, of which nearly 90% fall into non-protein-coding regions [1]. The recent publication of low-coverage sequencing data from large numbers of unrelated individuals offers a broad catalog of genetic variation in three major population groups that is complementary to deep sequencing of related individuals [2]. Recently, investigators used a family-sequencing approach to fine map recombination sites, and combined broad population genetic variation data with phased family variant data to identify putative compound heterozygous loci associated with the autosomal recessive Miller syndrome [3]. We previously developed and applied a methodology for interpretation of genetic and environmental risk in a single subject using a combination of traditional clinical assessment, whole genome sequencing, and integration of genetic and environmental risk factors [4]. The combination of these methods and resources and their application to phased genetic variant data from family based sequencing has the potential to provide unique insight into topology of genetic variation, haplotype information, and genetic risk.

One of the challenges to interpretation of massively parallel whole genome sequence data is the assembly and variant calling of sequence reads against the human reference genome. Although *de novo* assembly of genome sequences from raw sequence reads represents an alternative approach, computational limitations and the large amount of mapping information encoded in relatively invariant genomic regions make this an unattractive option presently. The National Center for Biotechnology Information (NCBI) human reference genome in current use [5] is derived from DNA samples from a small number of anonymous donors and therefore represents a small sampling of the broad array of human genetic variation. Additionally, this reference sequence contains both common and rare disease risk variants, including rare susceptibility variants for acute lymphoblastic leukemia and the Factor V Leiden allele associated with hereditary thrombophilia [6]. Thus, the use of the haploid NCBI reference for variant identification using high throughput sequencing may complicate detection of rare disease risk alleles. Furthermore, the detection of alternate alleles in high-throughput sequence data may be affected by preferential mapping of short reads containing the reference base over those containing an alternate base [7]. The effects of such biases on genotype accuracy at common variant loci remain unclear.

Here we report the development of a novel, ethnically concordant major allele reference sequence and the evaluation of its use in variant detection and genotyping at disease risk loci. Using this major allele reference sequence, we provide an assessment of haplotype structure and phased genetic risk in a family quartet with familial thrombophilia.

## Results

### Study subjects and genome sequence generation

**Figure 1:**
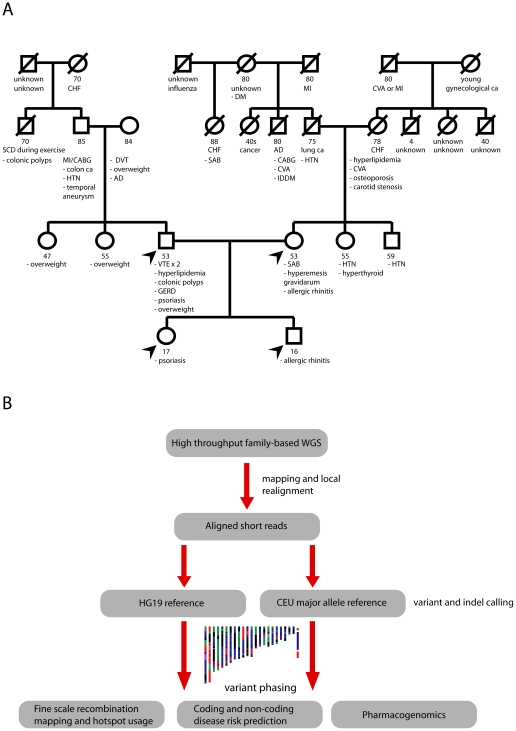
Pedigree and genetic risk prediction workflow. A, Family pedigree with known medical history. The displayed ages represent the age of death for deceased subjects or the age at the time of medical history collection (9/2010) for living family members. Arrows denote sequenced family members. Abbreviations: AD, Alzheimer's disease; CABG, coronary artery bypass graft surgery; CHF, congestive heart failure; CVA, cerebrovascular accident; DM, diabetes mellitus; DVT, deep venous thrombosis; GERD, gastroesophageal reflux disease; HTN, hypertension; IDDM, insulin-dependent diabetes mellitus; MI, myocardial infarction; SAB, spontaneous abortion; SCD, sudden cardiac death. B, Workflow for phased genetic risk evaluation using whole genome sequencing.

Clinical characteristics of the study subjects and the heuristic for the genome sequence generation and analysis are described in Figure 1. Two first-degree family members, including the father in the sequenced quartet, have a history of venous thrombosis; notably, the sequenced father has a history of recurrent venous thromboembolism despite systemic anticoagulation. Both parents self-reported northern European ancestry. We used the Illumina GAII sequencing platform to sequence genomic DNA from peripheral blood monocytes from four individuals in this nuclear family, providing 39.3x average coverage of 92% of known chromosomal positions in all four family members (Figure S1).

### Development of ethnicity-specific major allele references

**Figure 2:**
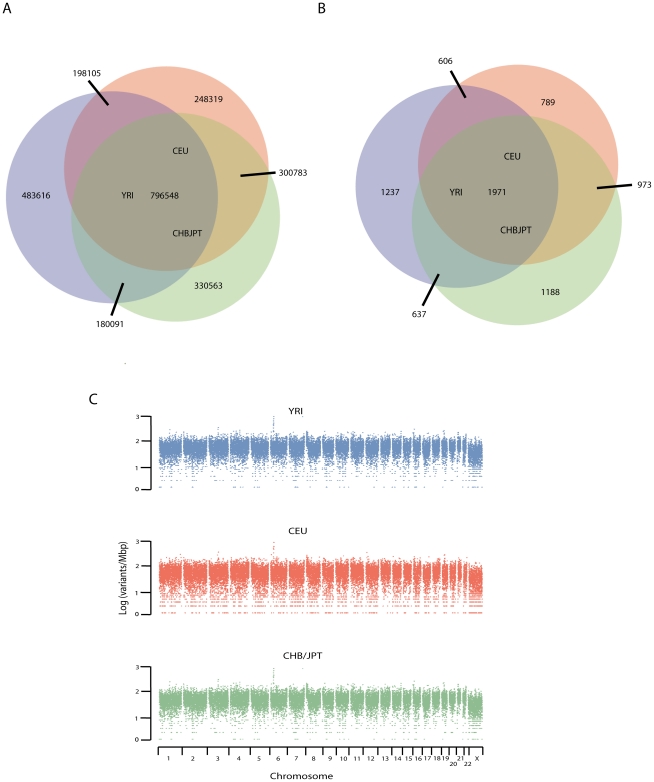
Development of major allele reference sequences. Allele frequencies from the low coverage whole genome sequencing pilot of the 1000 genomes data were used to estimate the major allele for each of the three main HapMap populations. The major allele was substituted for the NCBI reference sequence 37.1 reference base at every position at which the reference base differed from the major allele, resulting in approximately 1.6 million single nucleotide substitutions in the reference sequence. A, Approximately half of these positions were shared between all three HapMap population groups, with the YRI population containing the greatest number of major alleles differing from the NCBI reference sequence. B, Number of disease-associated variants represented in the NCBI reference genome by the minor allele in each of the three HapMap populations. C, Number of positions per Mbp at which the major allele differed from the reference base by chromosome and HapMap population.

We developed three ethnicity specific major allele references for European (European ancestry in Utah (CEU)), African (Yoruba from Ibadan, Nigereia (YRI)), and East Asian (Han Chinese from Beijing and Japanese from Tokyo (CHB/JPT)) HapMap population groups using estimated allele frequency data at 7,917,426, 10,903,690, and 6,253,467 positions cataloged in the 1000 genomes project. Though relatively insensitive for very rare genetic variation, the low coverage pilot sequencing data (which comprises the majority of population-specific variation data) has a sensitivity for an alternative allele of >99% at allele frequencies >10% and thus has high sensitivity for detecting the major allele [2]. Substitution of the ethnicity-specific major allele for the reference base resulted in single base substitutions at 1,543,755, 1,658,360, and 1,676,213 positions in the CEU, YRI, and CHB/JPT populations, respectively (Figure 2A). There were 796,548 positions common to all three HapMap population groups at which the major allele differed from the NCBI reference base. Variation from the NCBI reference genomes was relatively uniform across chromosomal locations with the exception of loci in and near the Human Leukocyte Antigen (HLA) loci on chromosome 6p21 (Figure 2C). Of variant positions associated with disease in our manually curated database of 16,400 genotype-disease phenotype associations, 4,339, 4,451, and 4,769 are represented in the NCBI reference sequence by the minor allele in the CEU, YRI, and CHBJPT populations, respectively (Figure 2B). There are 1,971 disease-associated variant positions represented on the NCBI reference sequence by the minor allele in all three population groups (Figure 2B). Of these manually-curated disease-associated variants, 23 are represented on the NCBI reference sequence by minor alleles with a frequencies of less than 5% in all three population groups, and 18 are represented by minor alleles with frequencies of less than 1% in at least one population group (Table S1).

To test the alignment performance of the major allele reference sequences, we performed alignments of one lane of sequence data to chromosome 6, which demonstrated the greatest population-specific divergence between the major allele in each HapMap population and the NCBI reference, and chromosome 22 in the NCBI and CEU major allele references (Table S2). These analyses demonstrated that ∼0.01% more reads mapped uniquely to the major allele reference sequence than to the NCBI reference sequence. We identified sequence variants in the family quartet by comparison with the HG19 reference as well the CEU major allele reference we developed, resulting in single nucleotide substitutions at an average distance of 699 base pairs when compared with the NCBI reference and 809 base pairs when compared with the CEU major allele reference. We also identified 859,870 indels at an average inter-marker distance of 3.6 kbp.

### A major allele reference sequence reduces genotyping error at variant loci associated with disease traits

Specific to the family quartet, of 16,400 manually-curated single nucleotide polymorphisms associated with disease traits, 10,396 were variant in the family when called against the NCBI reference genome, and 9,389 were variant in the family when called against the major allele reference genome. The genotyping error rate for these disease-associated variants, estimated by the Mendelian inheritance error (MIE) rate per variant, was 38% higher in the variants called by comparison with the NCBI reference genome (5.8 per 10,000 variants) than in variants called by comparison with the major allele reference genome (4.2 per 10,000 variants). There were 233 genotype calls at 130 disease-associated variant positions that differed across the quartet between the NCBI reference genome and the major allele reference genome (summary for each genome is provided in Table S3). Among these variants, 161/188 genotypes (85.6%) in the major allele call set were concordant with genotypes from orthogonal genotyping technology, whereas only 68/188 (36.2%) in the NCBI call set were concordant with independent genotyping.

### Inheritance state analysis identifies >90% of sequencing errors

**Figure 3:**
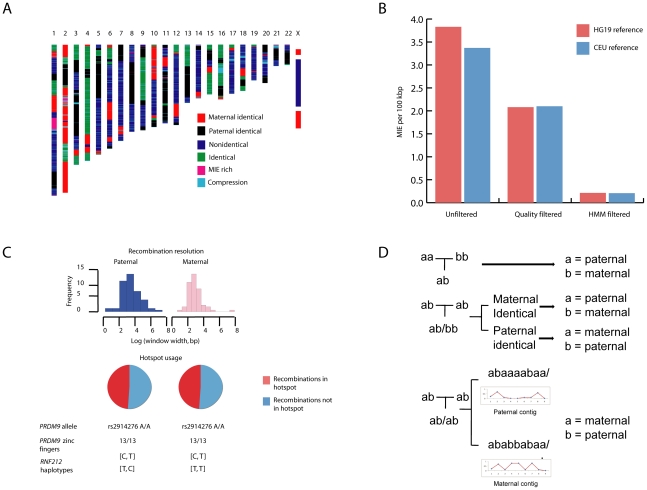
Inheritance state analysis, error estimation, and phasing. A, A Hidden Markov Model (HMM) was used to infer one of four Mendelian and two non-Mendelian inheritance states for each allele assortment at variant positions across the quartet. “MIE-rich” refers to Mendelian-inheritance error (MIE) rich regions. “Compression” refers to genotype errors from heterozygous structural variation in the reference or study subjects, manifest as a high proportion of uniformly heterozygous positions across the quartet. B, A combination of quality score calibration using orthogonal genotyping technology and filtering SNVs in error prone regions (MIE-rich and compression regions) identified by the HMM resulted in >90% reduction in the genotype error rate estimated by the MIE rate. C, Consistent with *PRDM9* allelic status, approximately half of all recombinations in each parent occurred in hotspots. The mother has two haplotypes in the gene *RNF212* associated with low recombination rates, while the father has one haplotype each associated with high and low recombination rates. Notation denotes base at [rs3796619, rs1670533]. D, Variant phasing using pedigree, inheritance state, and population linkage disequilibrium data. Pedigree data were first used to phase informative allele assortments in trios (top). The inheritance state of neighboring regions was used to phase positions in which all members of a mother-father-child trio were heterozygous and the sibling was homozygous for the reference or non-reference allele (middle). For uniformly heterozygous positions, we phased the non-reference allele using a maximum likelihood model to assign the non-reference allele to paternal or maternal chromosomes based on population linkage disequilibrium with phased SNVs within 250 kbp (bottom). In all panels *a* corresponds to the reference allele and *b* to the non-reference allele.

Sequencing family quartets allows for precise identification of meiotic crossover sites from boundaries between inheritance states and superior error control [3]. We resolved contiguous blocks of single nucleotide variants into one of four Mendelian inheritance states or two error states. Using this methodology, we identified 3.1% of variant positions as associated with error prone regions (Figure 3A). Using a combination of these methods and quality score calibration with orthogonal genotyping technology, we identified 94% of genotyping errors, with the greatest reduction in error rate resulting from filtering of variants in error prone regions (Figure 3B). We estimated a final genotype error rate by three methods of between 5.26×10^−7^, estimated by the state consistency error rate in identical-by-descent regions, and 2.1×10^−6^, estimated by the MIE rate per bp sequenced.

### Prior population mutation rate estimates are biased upwards by the reference sequence

After excluding variants in sequencing-error prone regions, we identified 4,302,405 positions at which at least one family member differed from the NCBI reference sequence and 3,733,299 positions at which at least one family member differed from the CEU major allele reference sequence (Figure S2). With respect to the NCBI reference sequence, this corresponds to an estimated population mutation rate (Watterson's θ [8]) of 9.2×10^−4^, matching previous estimates [3]. However, in comparison with the CEU major allele reference, we estimated a lower population mutation rate of 7.8×10^−4^, suggesting that previous estimates may have been biased upwards by comparison with the NCBI reference sequence.

### Male and female recombinations occur with nearly equal frequency in this family and approximately half occur in hotspots

Boundaries between contiguous inheritance state blocks defined 55 maternal and 51 paternal recombination events across the quartet at a median resolution of 963 base pairs. A parallel heuristic analysis of recombination sites confirmed our observation of nearly equal paternal and maternal recombination frequency (Figure 3C). Fine scale recombination mapping and long range phasing revealed that the mother has two haplotypes ([C, T] and [T, T]) at SNPs rs3796619 and rs1670533 that are associated with low recombination rates in females, while the father has one haplotype associated with low recombination rate in males [T, C] [9]. The father also has the common [C,T] haplotype which is associated with high recombination rates in males when compared with the other two observed haplotypes. We found that 25 of 51 paternal recombination windows (49%) and 27 of 55 maternal recombination windows (49%, Figure 3) were in hotspots (defined by maximum recombination rate of >10 cM/Mbp), while only ∼4 (4.1%) would be expected by chance alone (*p* = 2.0×10^−73^ for hotspot enrichment according to Monte Carlo permutation). Both parents carry 13 zinc finger repeats in the *PRDM9* gene (Entrez Gene ID 56979) and are homozygous for the rs2914276-A allele; both of these loci have been previously associated with recombination hotspot usage [10]–[13]. We used a combination of per-trio phasing, inheritance state of adjacent variants, and population linkage disequilibrium data to provide long range phased haplotypes (Figure 3D).

### Rare variant analysis identifies multi-genic risk for familial thrombophilia

It has been estimated from population sequencing data that apparently healthy individuals harbor between 50 and 100 putative loss of function variants in genes associated with Mendelian diseases and traits [2]. Many of these variants are of limited import, either because they result in subtle phenotypes or have no biological effect. Thus, prioritization of putative loss of function variants remains a significant challenge. We used a combination of manually-curated rare-variant disease risk association data, an internally-developed method for scoring risk variants according to potential clinical impact, and existing prediction algorithms [14], [15] (Figure S3 and Table S4) to provide genetic risk predictions for phased putative loss-of-function variants for the family quartet (Table 1). To further characterize the potential adverse effects of non-synonymous single nucleotide variants (nsSNVs), we implemented a multiple sequence alignment (MSA) of 46 mammalian genomes, described further in Text S1, that is similar to that implemented in the Genomic Evolutionary Rate Profiling score [16], [17]. For coding variants of unknown significance, the mammalian evolutionary rate is proportional to the fraction of selectively neutral alleles [18] and can therefore serve as a prior expectation in determining the likelihood that an observed nsSNV is deleterious.

**Table 1:** Putative loss of function variants across the family quartet.

	All variants		All rare/novel		Rare/novel and OMIM-disease associated gene	
Variant type	HG19 reference (n = 4302405)	CEU reference (n = 3733299)	HG19 reference (n = 351555)	CEU reference (n = 354074)	HG19 reference	CEU reference
Coding-missense	9468	7982	1276	1276	203	200
Coding-nonsense	52	50	13	13	1	1
Coding-synonyn	11663	9928	1061	1059	186	186
Intronic	1303341	1128283	116276	115397	19544	19766
Splice-5′	156	147	16	16	0	0
Splice-3′	98	96	9	9	1	1
UTR-5′	40142	37794	3637	3619	510	516
UTR-3′	61826	59396	5989	5953	848	857
miRNA target	0	0	0	0	0	0
Pri-miRNA	2	2	1	1	0	0
Mature miRNA	0	0	0	0	0	0
Coding indels	1519	1476	432	412	73	71
Coding frameshift indels	440	418	273	253	29	27

Abbreviations: CEU reference, variant calls against CEU major allele reference; HG19 reference, variant calls against NCBI reference sequence 37.1; miRNA, micro RNA; Pri-miRNA, primary microRNA transcript; OMIM, Online Mendelian Inheritance In Man database; UTR, un-translated region.

Of 354,074 rare or novel variants compared with the CEU major allele reference sequence, we identified 200 non-synonymous variants in coding regions, 1 nonsense variant, 1 single nucleotide variant (SNV) in the conserved 3′ splice acceptor dinucleotides, and 27 novel frame-shifting indels in genes associated with Mendelian diseases or traits. Consistent with our prior observations and a conserved regulatory role for microRNAs (miRNAs), we found no rare or novel SNVs in mature miRNA sequence regions or miRNA target regions in 3′ UTRs. There were four compound heterozygous variants in disease-related genes and three homozygous variants in disease-related genes (Table S6). Five variants across the family quartet are associated with Mendelian traits (Table 2). One variant in the gene *F5* (Entrez Gene ID 2153), encoding the coagulation factor V, confers activated protein C resistance and increased risk for thrombophilia [19], [20]. Another variant (the thermolabile C677T variant) in the gene *MTHFR* (Entrez Gene ID 4524), encoding methylenetetrahydrofolate reductase, predisposes heterozygous carriers to hyper-homocysteinemia and may have a synergistic effect on risk for recurrent venous thromboembolism [21], [22]. Follow-up serological analysis demonstrated the father's serum homocysteine concentration was 11.5 µmol/L (Table S11). We were able to exclude a homozygous variant in *F2* (Entrez Gene ID 2147), a gene known to be associated with hereditary thrombophilia, based on its high evolutionary rate in multiple sequence alignment (Table S5). It is likely that these variants in *F5* and *MTHFR* contribute digenic risk for thrombophilia passed to the daughter but not son from the father. This is consistent with the father's clinical history of two venous thromboemboli, the second of which occurred on systemic anticoagulation. The daughter has a third variant inherited from her mother, the Marburg I polymorphism, in the hyaluronan binding protein 2 (*HABP2*, Entrez Gene ID 3026) gene known to be associated with inherited thrombophilia, thus contributing to multigenic risk for this trait [23]–[25]. Thus, our prediction pipeline recapitulated multigenic risk for the only manifest phenotype, recurrent thromboembolism, in the family quartet and provided a basis for a rational prescription for preventive care for the daughter.

**Table 2:** Rare variants with known clinical associations.

Chromosome	Gene	rsid	Affected family members	Disease	Inheritance	Onset-earliest	Onset-median	Severity	Actionability	Lifetime risk	Variant pathogenicity
12	*VWF*	rs61750615	M, S, D	Von Willebrand disease	Incomplete dominant	1	1	5	5	variable	7
10	*HABP2*	rs7080536	M, S, D	Carotid stenosis, thrombophilia	AD	4	4	1	5	variable	7
19	*SLC7A9*	rs79389353	M, D	Cysteinuria – kidney stones	AR	1	1	3	5	7	7
1	*F5*	rs6025	F, D	Thrombophilia	Incomplete dominant	4	4	4	5	2	7
1	*MTHFR*	rs1801133	F, D	Hyperhomocystein-emia	AR	1	1	1	6	2	7

Key: Father, mother, son, daughter  = F, M, S, D. Abbreviations: AD, autosomal dominant; AR, autosomal recessive. Variants were scored according to disease phenotype features and variant pathogenicty as outlined in Table S4.

Association between synonymous SNVs (sSNVs) and disease has recently been described [26]. sSNVs may affect gene product function in several ways, including codon usage bias, mRNA decay rates, and splice site creation and/or disruption (Figure S4). We developed and applied an algorithm (Text S1), for predicting loss of function effects of 186 rare and novel sSNVs in Mendelian disease associated genes based on change in mRNA stability, splice site creation and loss, and codon usage bias. We found that one sSNV in the gene *ATP6V0A4* (Entrez Gene ID 50617) was predicted to significantly reduce mRNA stability, quantified by the change in free energy in comparison with the reference base at that position (Figure S5). Further secondary structure prediction demonstrated that this SNV likely disrupts a short region of self-complementarity that forms a stable tetraloop (Figure S5) in the resultant mRNA. Homozygosity for loss of function (largely protein truncating) variants in this gene is associated with distal renal tubular acidosis, characterized by metabolic acidosis, potassium imbalance, urinary calcium insolubility, and disturbances in bone calcium physiology [27].

### Common variant risk prediction identifies risk for obesity and psoriasis

**Figure 4:**
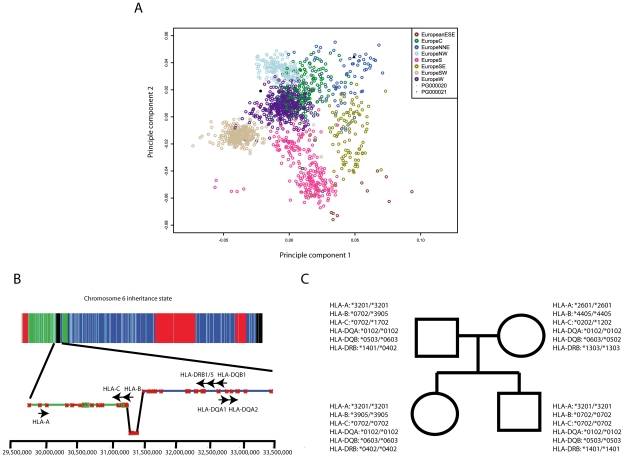
Ancestry and immunogenotyping using phased variant data. A, Ancestry analysis of maternal and paternal origins based on principle components analysis of SNP genotypes intersected with the Population Reference Sample dataset. B, The HMM identified a recombination spanning the HLA–B locus and facilitated resolution of haplotype phase at HLA loci. Contig colors in the lower panel correspond to the inheritance state as depicted in Figure 3A. C, Common HLA types for family quartet based on phased sequence data.

Results from Genome Wide Association Studies (GWAS) provide a rich data source for assessment of common disease risk in individuals. To provide a population risk framework for genetic risk predictions for this family quartet, we first localized ancestral origins based on principal components analysis of common single nucleotide polymorphism (SNP) data in each parent and the Population Reference Sample (POPRES) dataset [28] (Figure 4A). This analysis demonstrated North/Northeastern and Western European ancestral origins for maternal and paternal lineages, respectively.

**Figure 5:**
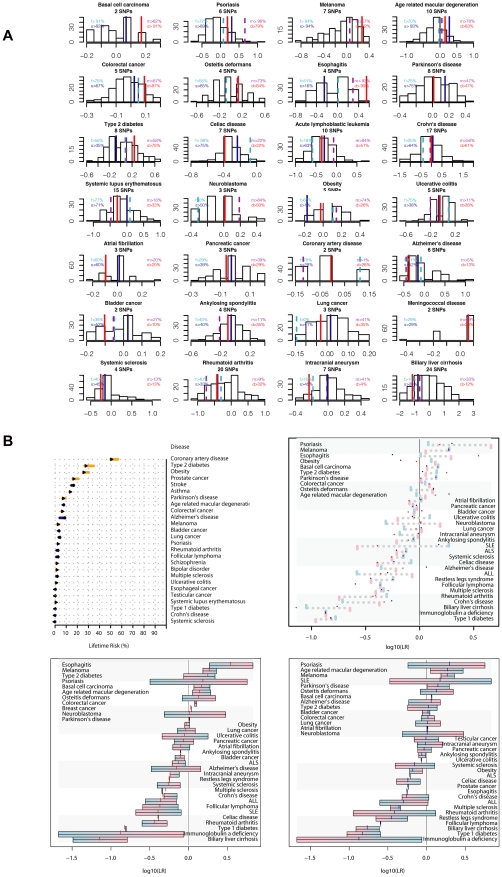
Common variant risk prediction. A, Common variant risk prediction for 28 disease states for each of the family members (f, father; m, mother; s, son; d, daughter) and 174 ethnicity-matched HapMap subjects. The x-axis in each plot represents the log10(likelihood ratio) for each disease according to allelic distribution of SNPs identified in the literature as significantly associated with disease by 2 or more studies including 2000 or more total subjects. B, Upper left: pre (base) and post (bar end) estimates of disease risk for the father according to common variant risk prediction, derived from the pre-probability of disease multiplied by the composite likelihood ratio from all SNPs meeting the criteria described above. Upper right: Composite likelihood ratio estimates for disease risk according to common genetic variation. Blue bars represent paternal estimate, pink bars represent maternal estimate, red points represent the estimate for the daughter, and blue points represent the estimate for the son. Lower panels: parental haplotype contribution to disease risk for each child (points) for the daughter (lower left) and son (lower right). Blue shading represents paternal haplotype risk allele contribution and pink shading represents maternal haplotype risk allele contribution.

HLA groups are associated with several disease traits and are known to modify other genotype - disease trait associations [29]–[31]. We used long-range phased haplotypes and an iterative search (described in full in Text S1) for the nearest HLA tag haplotype [32] to provide HLA types for each individual prior to downstream risk prediction (Figure 4B and 4C). We then calculated composite likelihood ratios (LR) for 28 common diseases for 174 ethnically-concordant HapMap CEU individuals, and provided percentile scores for each study subject's composite LR for each disease studied (Figure 5A). All four family members were at high risk for psoriasis, with the mother and daughter at highest risk (98^th^ and 79^th^ percentiles, respectively). We also found that both parents were predisposed to obesity, while both children had low genetic risk for obesity. Discordant risks for common disease between parents and at least one child were also seen for esophagitis and Alzheimer's disease. Phased variant data were further used to provide estimates of parental contribution to disease risk in each child according to parental risk haplotypes (Figure 5B).

### Pharmacogenomic variant annotation informs thrombophilia therapeutics

We used the Pharmacogenomics Knowledge Base (PharmGKB, http://www.pharmgkb.org/) to evaluate associations between phased variants and 141 drugs (Tables S8, S9, S10). In light of the family history of venous thrombosis and the father's use of the anticoagulant warfarin, we focused particularly on relationships between SNPs and anticoagulant and antiplatelet agents. All family members are homozygous for the most common *CYP2C9* allele (CYP2C9*1, Table 3) associated with normal warfarin pharmacokinetics, and heterozygous for the allele at VKORC1-1639 (rs9923231) associated with therapeutic prolongation of the international normalized ratio at low doses of warfarin. We used this variant data and clinical data to predict the father's exact empirically-determined dose of warfarin (5 mg) using the International Warfarin Pharmacogenetics Consortium dosing algorithm [33].

**Table 3:** Drug metabolizing enzyme variants.

		Father		Mother		Sister		Brother	
Drug Metabolizing Enzyme	Drugs Metabolized	Genotype	Phenotype	Genotype	Phenotype	Genotype	Phenotype	Genotype	Phenotype
CYP2C9*	warfarin,NSAIDS (naproxen, ibuprofen, celecoxib, etc.), sulfonylureas (glimepiride, glipizide, etc.) fluvastatin	*1/*1	normal metabolizer	*1/*1	normal metabolizer	*1/*1	normal metabolizer	*1/*1	normal metabolizer
CYP2C19†	clopidogrel, proton pump inhibitors (omeprazole, pantoprazole, etc.), citalopram	*17/*2	Undetermined‡: 1 ultra metabolizer allele and 1 loss-of-function allele	*17/*17	ultra metabolizer	*17/*17	ultra metabolizer	*17/*2	Undetermined‡:1 ultra metabolizer allele and 1 loss-of-function allele
CYP2D6§	codeine, metoprolol, tamoxifen, fluoxetine	*1/*4	intermediate metabolizer	*1/*1	normal metabolizer	*1/*4	intermediate metabolizer	*1/*4	intermediate metabolizer

*CYP2C9 genotypes checked and ruled out: *2, *3, *5, *8, *9, *10, *11, *12, *18; absence of these alleles defaults to *1.

**†:** CYP2C19 genotypes based on single defining SNPs for the *17 and *2 alleles; all other alleles ruled out by default.

**‡:** The *in vivo* phenotype for the combination of an increased activity allele and a loss-of-function allele for CYP2C19 is not well studied to date. According to Scott et al [53], one paper has reported intermediate activity for this allele combination with respect to clopidogrel, but the study was not replicated and therefore the phenotype is considered provisional. The actual phenotype associated with this combination may vary depending upon other factors such as the medication(s) the patient is taking, as well as other inducers and inhibitors of CYP2C19.

**§:** CYP2D6 genotypes checked: *2, *4, *5, *10, *15, *8, *11, *12, *14, *17, *19, *20, *29, *31, *35, *40, *41, *69; absence of these alleles defaults to *1.

We also found that the mother and daughter are both homozygous for an ultra-rapid metabolism allele at *CYP2C19* (Entrez Gene ID 1557), which encodes a key metabolizer of the pro-drug clopidogrel, an antiplatelet agent used in the prevention and therapy of cardiovascular disease. Because the metabolic activity of CYP2C19 is directly correlated with the antiplatelet activity of clopidogrel, there is a higher bleeding risk associated with clopidogrel use in the mother and daughter. This finding has significant implications for the daughter, who has multigenic risk for thrombophilia and may require anticoagulant therapy should she develop thrombosis; concomitant use of clopidogrel in this setting may contribute further to bleeding risk associated with systemic anticoagulation. Full details of pharmacogenetic variants in other key metabolic enzymes and associated pharmacokinetic and pharmacodynamic profiles are displayed in Table 4.

**Table 4:** Genetic pharmacological response predictions.

SNP location	Drug(s)	Drug(s) more likely to work	Drug(s) less likely to work	Drug(s) more likely to cause side effect	Drug(s) less likely to cause side effect	Drug dose(s) above average	Drug dose(s) below average	Drug dose(s) average	No PGx action/ phenotype unknown	Confidence level
rs9934438	warfarin							F, M, S, D		High
rs1954787	citalopram	F, M, D	S							High
rs776746	cyclosporine						F, M, S, D			High
rs1800460	thiopurines									High
rs2108622	warfarin						F, M, S, D			Medium
rs4680	morphine					F, M, S, D				Medium
rs5443	statins	F, M	S, D							Medium
rs4253778	beta blocking agents	D	F, M ,S,							Medium
rs622342	metformin	M, S	F, D							Medium
rs7569963	citalopram			S	F				M, D	Medium
rs8012552	ACE inhibitors				F, M, S, D					Low
rs11209716	ACE inhibitors				F, S, D				M	Low

Key: Father, mother, son, daughter  = F, M, S, D. Abbreviations: ACE, angiotensin converting enzyme; PGx, pharmacogenomic. Family members' genotypes are compared to other possible genotypes; this is not a population-based statistic.

## Discussion

Here we describe phased genetic risk assessment in a family quartet using whole genome sequencing and an ethnicity-specific major allele reference. In doing so we present several novel findings: the development and application of ethnicity-specific major allele reference sequences; the definition of meiotic crossover sites to a median resolution of <1000 base pairs; the development of a computational algorithm to achieve genome-wide long-range haplotype phasing; the application of this phasing to HLA typing and clinical interpretation of genomes from a family quartet using internally developed databases of all publicly accessible genotype-phenotype association data; the demonstration and quantification of discordance of inherited disease risk within a nuclear family; and the successful prediction of the only manifest disease phenotype in this family.

At ∼1.6 million genomic positions, we found that the NCBI reference sequence is represented by the minor allele in each of the three HapMap populations, and over 4,000 of these variant loci are associated with disease traits. Despite only a small difference in the number of mapped reads between the major allele and NCBI reference sequences, we demonstrated that the use of a major allele reference reduces genotyping error at common disease-associated variant loci by ∼40%. Accordingly, among genotypes at 130 disease-associated variant loci that differ between the major allele reference and NCBI reference call sets, 85% of genotype calls made against the major allele reference were confirmed by orthogonal genotyping, whereas only 36% of calls made against the NCBI reference genome were concordant with orthogonal genotyping. These results suggest that there is genotyping bias introduced by the use of a non-major allele at common variant positions. This issue, which may be compounded by read-mapping reference biases [7], may be of particular importance in lower coverage depth sequencing projects, in which fewer sequence reads are used to generate genotype likelihoods for variant loci. Additionally, rare variants associated with larger disease risk, including the rare variant in *F5* associated with hereditary thrombophilia in this family, are represented by the minor allele on the haploid reference sequence at 23 positions. These estimates are only for curated disease associations in the published literature; the true number of rare disease risk alleles represented on the human reference genome is likely higher. The use of this reference sequence for whole genome sequence variant identification will not result in variant calls at these positions if the subjects being sequenced are homozygous for the risk allele. Using the major allele reference sequence and genome sequence variation data from a family quartet, we also provide new estimates for the population mutation rate that illustrate the upward bias in prior estimates that were derived via comparison with the NCBI human reference genome.

The incorporation of variant phase information into genetic risk prediction for common disease traits has several important advantages. HLA types are associated with several disease traits [29], [30], [34], contribute a major fraction of disease risk in autoimmune diseases [31], and are major factors in determining solid organ and tissue transplant compatibility. Classical methods for HLA typing are expensive and time consuming. Human leukocyte antigen (HLA) group typing from high-throughput short read sequence data has previously been challenging due to the high recombination frequency on chromosome 6 that complicates phasing, as well as the extreme genetic diversity in the HLA loci. Our approach that incorporates fine-mapping of recombination events with long-range phasing from polymorphic markers specific to the ethnic background of the sequenced family (i.e., variants called against the CEU major allele reference) simplifies the task of identifying tag haplotypes from high-throughput sequence data.

Furthermore, several common SNPs have been associated with disease risk, most notably diabetes mellitus 2, in a sex specific manner, such that maternal origin confers direct association with risk of disease and paternal origin confers indirect association with disease risk [35]. Our methods for determining parental origin of risk alleles allows for precise risk assessment utilizing this information as a prior, in contrast to un-phased genotype data.

Lastly, there is recent evidence that genetic risk scores based on common variants can provide disease risk estimates that are independent of family history [36]. Our phased risk estimates complement these findings by providing, for the first time, a fine map of the origins of discordant risks for common disease between generations in a nuclear family, demonstrating discordant parental-child disease risks for three common disease traits. Such methods serve to underline the additional power provided by sequencing over family history alone in providing more precise estimates of the inherited risk for individuals.

Family quartet sequencing allowed us to provide a fine map of meiotic crossover sites to sub-kb resolution, and we found that meiotic crossovers happen with nearly equal frequency in male and female parents in this family quartet. This is in contrast to previous observations in a family quartet and in mice that recombinations occur with greater frequency in females than in males [3], [37], [38]. Recent estimates of human sex-specific recombination rates have, however, demonstrated significant variation in recombination number, particularly in females [13], [39]–[41]. Two SNPs (rs3796619 and rs1670533), separated by 17 kb in the gene *RNF212* (Entrez Gene ID 285498), form a haplotype that is significantly associated with genome wide recombination rate in a sex-specific manner, such that the haplotype associated with the highest recombination rate in males is associated with a low female recombination rate. Notably, the *RNF212* haplotypes revealed by long range phasing in this quartet are associated with lower than average female and average male recombination rates [9]. These haplotype combinations likely contribute to below average recombination rate in the mother and average recombination rate in the father, potentially explaining our observations.

In this family quartet we observed that approximately half of recombinations occurred in previously identified hotspots. Hotspot usage in humans is variable, and is largely associated with allelic status at several loci in the vicinity of *PRDM9* [10]–[13]. The heritability for hotspot use explained by this locus is among the highest of all described quantitative trait loci and is determined by both single nucleotide substitutions in and near *PRDM9* and the number of zinc finger α-helices in exon 12 of the gene [13]. The use of whole genome sequencing allows for fine mapping of these sites and investigation of the relationship between *PRDM9* haplotypes and hotspot usage. Of the SNPs near *PRDM9*, rs2914276 is most significantly associated with hotspot usage heritability. In this quartet both parents are homozygous for the rs2914276-A allele that is associated with high hotspot usage as well as the number of zinc finger repeats in *PRDM9* [13].

We leveraged the power of sequencing nuclear families for identification of >90% of sequencing errors in the quartet, providing unprecedented accuracy of sequence information used for genetic risk interpretation and identification of compound heterozygous and multigenic disease risk. This approach, first applied to a family quartet in which two family members had Miller syndrome and ciliary dyskinesia [3], was extended into a tool for phasing genetic variants. We also applied algorithms for multiple sequence alignment for novel and rare nonsynonymous variant risk prediction, and functional prediction for the effects of synonymous SNPs. In doing so we identified multigenic risk for thrombophilia in the father and daughter, consistent with a history of recurrent venous thromboembolism in the father despite systemic anticoagulation. Notably, because the haploid human reference genome contains the Factor V Leiden mutation, if any family member had been homozygous for the Factor V Leiden mutation, single genome analysis using the NCBI reference sequence would not have identified this variant. This multigenic risk for thrombophilia is more consistent with the father's clinical history of recurrent thromboembolism on systemic anticoagulation than monogenic risk conferred by heterozygous factor V Leiden alone [42]. Furthermore, multigenic risk for thrombophilia identified in the daughter prior to first venous thrombosis has significant clinical implications in terms of risk mitigation.

Many challenges to interpretation of whole genome sequencing remain, both scientific and ethical [43], [44]. Sequencing error, reference sequence bias, lack of accurate information regarding haplotype phase, and lack of variant level annotation are several scientific challenges. The ethical challenges include privacy and confidentiality concerns, and legal, social, and insurance ramifications associated with acquiring and divulging comprehensive genetic information to research subjects or the general population. This information may reveal unanticipated risk for inherited disease traits that in some cases is based on incomplete or incorrectly annotated genotype-phenotype association data [45]. Whole genome re-sequencing will also identify new variants of unknown significant in genes associated with disease traits, potentially triggering expensive secondary testing [46]. Furthermore, though there is evidence that divulging genetic disease risk to unselected populations is not associated with short term psychological risk or decrement in quality of life metrics in a research setting [47], [48], it is not clear that the current genetic counseling workforce will have the capacity to deliver genetic data and counsel individuals if whole genome re-sequencing becomes widely available to the general population [43].

As technological advances lower the financial costs and time associated with generating whole genome sequence data, our ability to appropriately interpret these data must advance in step. The ethnicity-specific, family-based approaches to interpretation of genetic variation presented here are emblematic of the next generation of genetic risk assessment using whole genome sequencing.

## Materials and Methods

### Ethics statement

The study was approved by the Stanford University Institutional Review Board and all study subjects attended genetic counseling and provided informed written consent (or assent, in the case of the children). This consent process occurred at two points in time: before the sequencing was performed (overseen by Illumina, Inc., and conducted with a clinical geneticist) and before this clinical interpretation was performed (conducted with a genetic counselor, a research assistant, and a physician). Pedigree and genotyping results were discussed in a genetic counseling session in the context of information that may be obtained in clinical interpretation of genome sequence data and the personal and family risks and benefits that may arise in obtaining this information [43].

### DNA sequence generation

Genomic DNA was extracted from peripheral blood from the study subjects and sequenced using reversible terminator massively parallel sequencing on the GA II instrument at Illumina, Inc (San Diego, CA). Seventy-five base pair paired-end reads were mapped to the NCBI human reference genome 37.1 (HG19) using BWA software version 0.5.8a [49] with local realignment around known indels performed by the Genome Analyis Tool Kit (GATK) [50]. A total of 5.98 billion sequence reads mapped uniquely to the reference sequence across the quartet, resulting in 448 gigabases of sequence data. Variant calling was performed using SAMtools multi-sample pileup and BCFtools by comparison with HG19 and the CEU major allele reference sequence. Major allele reference sequences for the CEU, YRI, and CHB/JPT populations are available at http://datadryad.org/.

### Inheritance state determination and recombination mapping

We built on the inheritance state analysis algorithm developed by Roach, et al [3], to resolve contiguous blocks of SNVs into one of four Mendelian inheritance states using a Hidden Markov Model: paternal identical, in which each child receives the same allele from the father but different alleles from the mother; maternal identical, in which each child receives the same allele from the mother but different alleles from the father; identical; and nonidentical. Two additional non-Mendelian inheritance states were modeled (compression and Mendelian inheritance error (MIE) rich, described in Text S1 [3]). The modeling of two additional error states allowed for identification of error-prone regions that are difficult to sequence or properly map and genotype. After excluding error prone regions that are potential sources of spurious recombination site inferences, we re-analyzed the variant allele assortments using only four Mendelian inheritance states, identifying meiotic crossover windows as intervals between SNVs defining the end and start, respectively, of contiguous inheritance state blocks.

### Phasing

We applied a combination of per-trio pedigree information, inheritance state information, and population linkage disequilibrium data, described in full in Text S1, to provide long-range phasing of each of the four family members (Figure 3D). Briefly, we resolved phase of heterozygous variants in the children by: 1) the inheritance state of the surrounding variants in contiguous inheritance blocks (for variant positions at which each of three individuals in a father-mother-child trio was heterozygous for a non-reference allele and the sibling was homozygous for the reference or non-reference allele); 2) maximization of aggregate r^2^ from pair-wise pre-computed population linkage disequilibrium data from the SNP Annotation and Proxy Search (SNAP) database [51]. Phasing was performed for each adult in contigs according to passage of allele contigs to one, both, or neither of the children.

### Immunogenotyping

We used an iterative, leave-one-out heuristic search (Text S1) for the nearest tag haplotype for common HLA types [32] using phased variant data, assigning an HLA type to each chromosome for each study subject based on this nearest tag haplotype.

### Rare variant prioritization

We used a combination of prediction algorithms based on characteristics of amino acid change and predicted protein structural and functional changes (SIFT, Polyphen2) [14], [15], and a novel MSA of 46 mammalian species, in which we computed the evolutionary rate and time span at each genomic position according to the method of Fitch [52], to provide genetic risk predictions about non-synonymous coding variants in Mendelian-disease associated genes. These variants were further manually annotated according to disease phenotype features and variant pathogenicity as described in Table S4. Methods for functional prediction of codon usage bias, splice site disruption, and mRNA stability for synonymous coding variants, and annotation of variants in important non-coding regions are described in Text S1.

### Common variant risk prediction

We have developed a manually curated database of greater than 4000 publications investigating associations between 35,997 SNPs and 1,194 diseases or traits. We applied a combinatorial approach for point estimation of likelihood ratios of disease-SNP association, and generated composite likelihood ratios for groups of SNPs and associated diseases as described previously [4] from phased genetic variant data (described in full in the Text S1). In this analysis we included disease-SNP associations replicated in greater than 2 genome wide association studies with a total sample size of greater than 2000 individuals and only SNPs genotyped in the HapMap CEU population to provide a population-risk framework for interpreting composite likelihood ratios.

### Pharmacogenomics

We compiled 432 clinical annotations between 298 SNPs and drugs (Pharmacogenomics Knowledge Base, PharmGKB, http://www.pharmgkb.org/). For all family members in the quartet we evaluated associations between 248 phased SNPs, including 147 heterozygous loci, and 141 drugs (example annotation in Table S7, variant summary annotations in Tables S8, S9, S10). A full description of pharmacogenomic methods is found in Text S1.

## Supporting Information

Figure S1 Genotype coverage in quartet subjects. Paired end short reads were mapped to NCBI reference genome 37.1 as described in Text S1. A, Percentage of total chromosome length (including positions not covered by the reference sequence) successfully genotyped in all four individuals in the family quartet. Chromosome 23  =  X chromosome, chromosome 24  =  Y chromosome. B, Haploid depth of coverage by chromosome and individual at each successfully genotyped position. PG20  =  mother, PG21  =  father, PG22  =  son, PG23  =  daughter.
(TIF)

Figure S2 Variant types and error rate estimates for variants against NCBI reference 37.1 and CEU major allele reference. After short read mapping and local realignment, variants were called against the NCBI reference genome 37.1 and the CEU major allele reference. We first filtered likely spurious variant calls by mapping quality, read depth and genotyping quality. The inheritance state for all allele assortments was determined by HMM and error prone regions (compression regions and Mendelian inheritance error rich (MIE)-rich regions, which represent likely sequencing errors) were identified and excluded. A, We identified 606,757 fewer variants when compared the CEU major allele reference than the NCBI reference genome 37.1 (HG19 reference). B,C, Approximately 8% and 9% of variants called against the HG19 reference (B) and CEU major allele reference (C) were rare (allele frequency <5%) or novel (not found in dbSNP or 1000 genomes pilot project data), respectively.
(TIF)

Figure S3 Search heuristic for rare and novel variants. We first identified rare (allele frequency <5%) and novel variants (not found in dbSNP 132 or the august 2010 release of the 1000 genomes pilot data). We used the CCDS collection of coding sequences to assign rare and novel variants to coding and noncoding categories and annotated putative rare and novel loss of function variants in coding and noncoding regions of genes known to be associated with Mendelian diseases as defined by the Online Mendelian Inheritance in Man database. This list of variants was manually curated for association with known clinical syndromes and variant pathogenicity and phenotype information were scored as in Table S4.
(TIF)

Figure S4 Synonymous variant risk prediction. Three models for the association between a synonymous SNVs and gene function. A, Shifts in signal to noise ratios between energies of a window of nucleotides that surround the SNV locus. The random background model is generated as sequences that have identical nucleotide composition except for a small interval that contains the SNV locus, thus measuring the contribution of the reference and polymorphic nucleotide to mRNA free energy, which is used as a proxy of mRNA stability. B, Codon usage frequencies correlate with ribosome latency and have been shown to affect, sometimes dramatically, protein elongation dynamics. Codons are clustered based on their position and usage frequencies, in both the reference and SNV-containing transcript. Changes in cluster centroids are given as a measure of local influences of codon frequency changes to global codon usage structure. C, Splicing site generation or disruption is measured as the change in predicted odds ratio of a maximum entropy splicing model. All synonymous SNVs were analyzed using these three criteria.
(TIF)

Figure S5 Synonymous SNV prediction identifies a putative loss of function variant in ATP6V0A4. A, A *Z* score method for predicting free energy change conferred by synonymous single nucleotide variants identified loci in the coding region of *ATP6V0A4* associated with a significant change in mRNA free energy. B, Predicted change in mRNA secondary structure by C>T transition at rs74921348.
(TIF)

Table S1 Very rare (minor allele frequency <1%) disease risk alleles in the NCBI reference genome.
(DOC)

Table S2 BWA alignment efficiency using HG19 and the CEU major allele reference genomes.
(DOC)

Table S3 Genotype changes for disease-associated variants using a major allele reference sequence.
(DOC)

Table S4 Prioritization scheme for rare and novel single nucleotide variants.
(DOC)

Table S5 Variants of potential significance in OMIM-curated disease associated genes.
(DOC)

Table S6 Compound heterozygous and homozygous variants in Mendelian disease associated genes.
(DOC)

Table S7 Example pharmacogenomics annotation.
(DOC)

Table S8 Variants associated with drug efficacy.
(DOC)

Table S9 Variants associated with adverse drug response.
(DOC)

Table S10 Variants associated with drug dosing.
(DOC)

Table S11 Laboratory assessment of the father.
(DOC)

Text S1 Supplementary materials and methods.
(DOC)
