# Supplementary figures and images for: Phased Whole-Genome Genetic Risk in a Family Quartet Using a Major Allele Reference Sequence
Source: PLoS Genet. 2011 Sep 15;7(9):e1002280. doi: 10.1371/journal.pgen.1002280 (PMC3174201; doi:10.1371/journal.pgen.1002280)

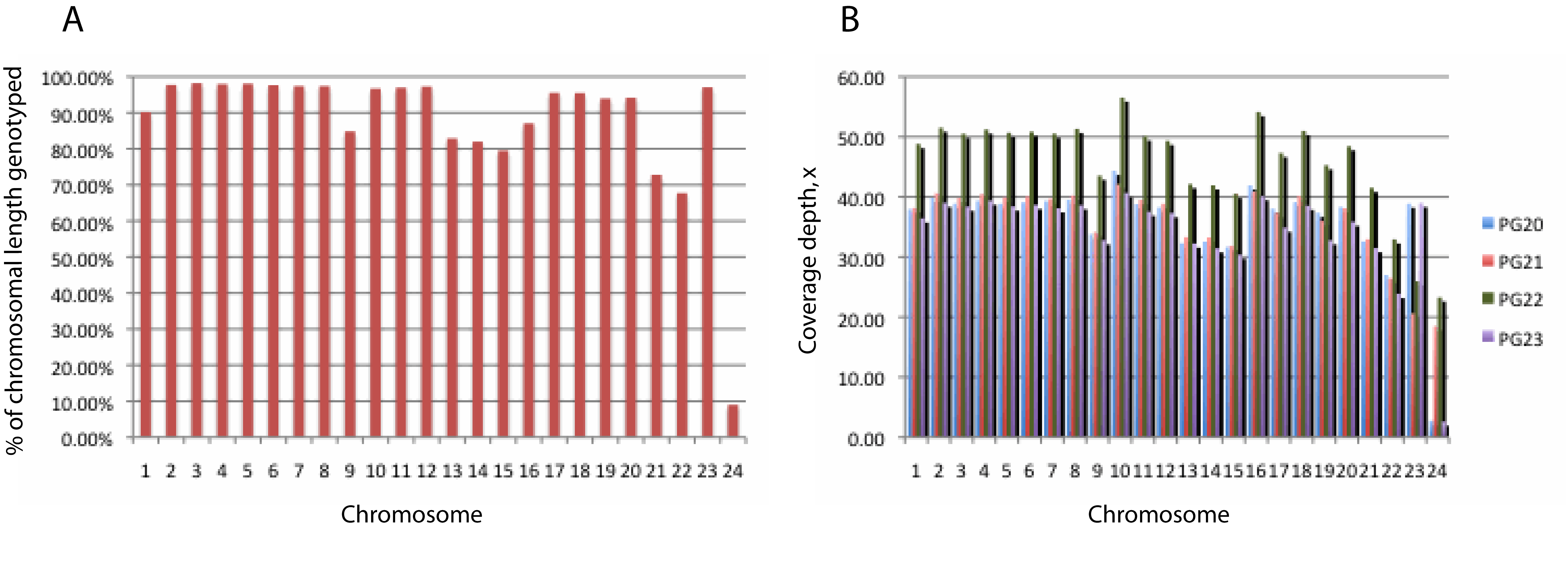

Supplement: Figure S1 — Genotype coverage in quartet subjects. Paired end short reads were mapped to NCBI reference genome 37.1 as described in Text S1. A, Percentage of total chromosome length (including positions not covered by the reference sequence) successfully genotyped in all four individuals in the family quartet. Chromosome 23 = X chromosome, chromosome 24 = Y chromosome. B, Haploid depth of coverage by chromosome and individual at each successfully genotyped position. PG20 = mother, PG21 = father, PG22 = son, PG23 = daughter. (TIF) [file pgen.1002280.s001.tif]

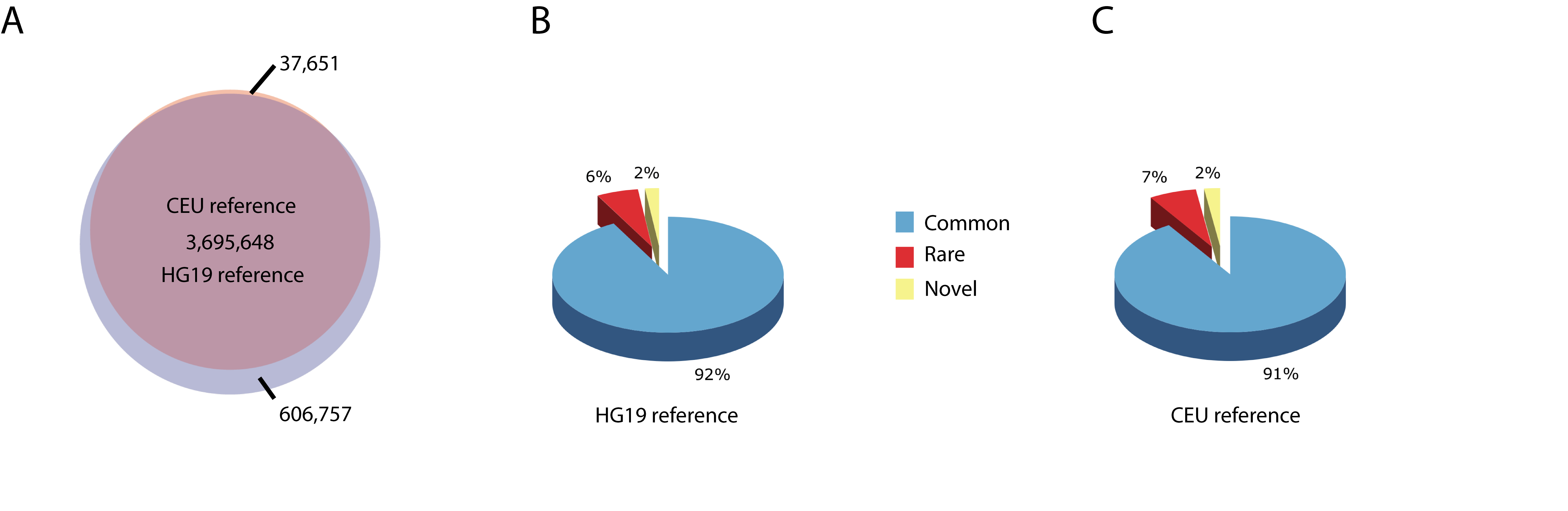

Supplement: Figure S2 — Variant types and error rate estimates for variants against NCBI reference 37.1 and CEU major allele reference. After short read mapping and local realignment, variants were called against the NCBI reference genome 37.1 and the CEU major allele reference. We first filtered likely spurious variant calls by mapping quality, read depth and genotyping quality. The inheritance state for all allele assortments was determined by HMM and error prone regions (compression regions and Mendelian inheritance error rich (MIE)-rich regions, which represent likely sequencing errors) were identified and excluded. A, We identified 606,757 fewer variants when compared the CEU major allele reference than the NCBI reference genome 37.1 (HG19 reference). B,C, Approximately 8% and 9% of variants called against the HG19 reference (B) and CEU major allele reference (C) were rare (allele frequency <5%) or novel (not found in dbSNP or 1000 genomes pilot project data), respectively. (TIF) [file pgen.1002280.s002.tif]

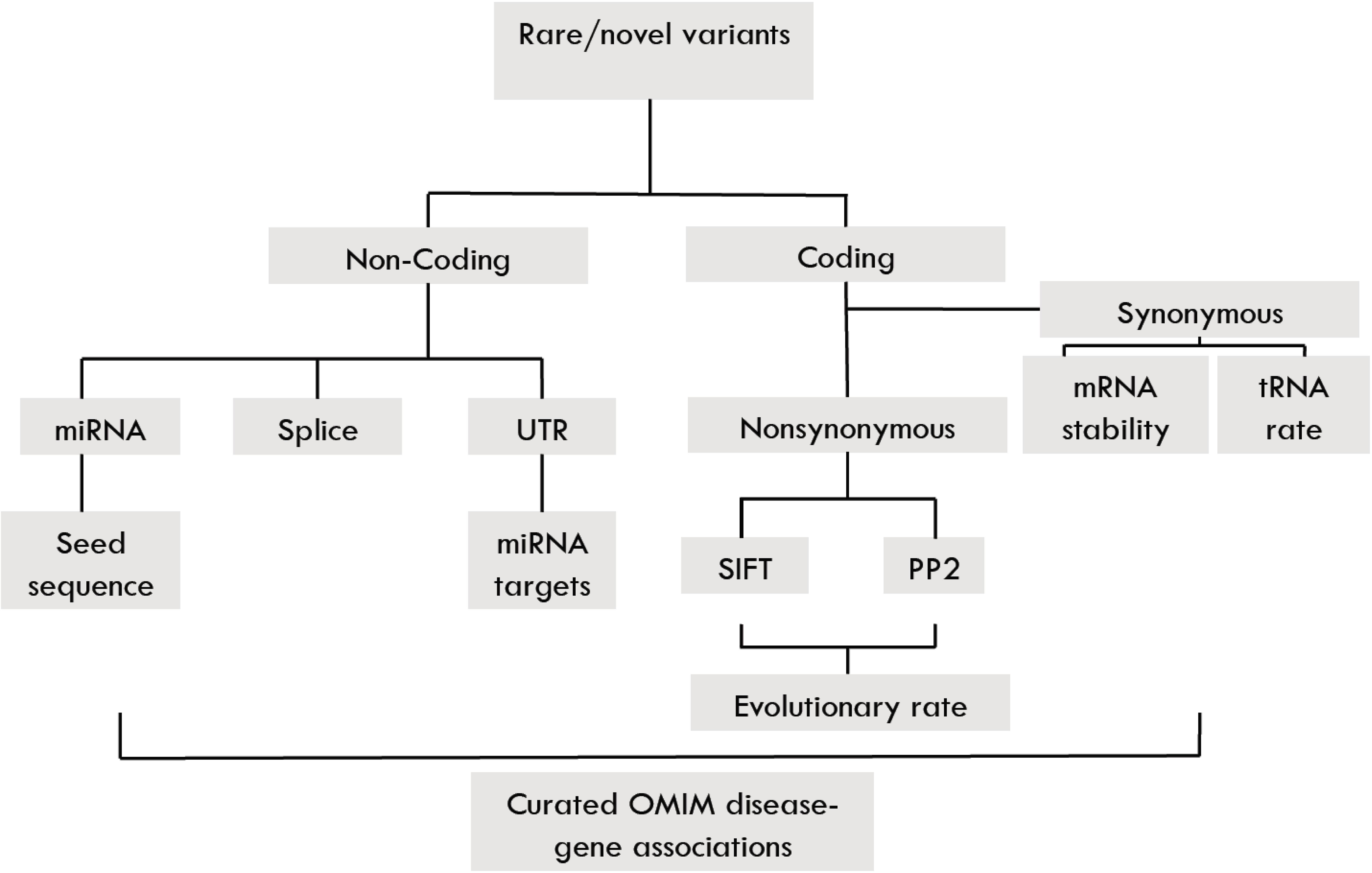

Supplement: Figure S3 — Search heuristic for rare and novel variants. We first identified rare (allele frequency <5%) and novel variants (not found in dbSNP 132 or the august 2010 release of the 1000 genomes pilot data). We used the CCDS collection of coding sequences to assign rare and novel variants to coding and noncoding categories and annotated putative rare and novel loss of function variants in coding and noncoding regions of genes known to be associated with Mendelian diseases as defined by the Online Mendelian Inheritance in Man database. This list of variants was manually curated for association with known clinical syndromes and variant pathogenicity and phenotype information were scored as in Table S4. (TIF) [file pgen.1002280.s003.tif]

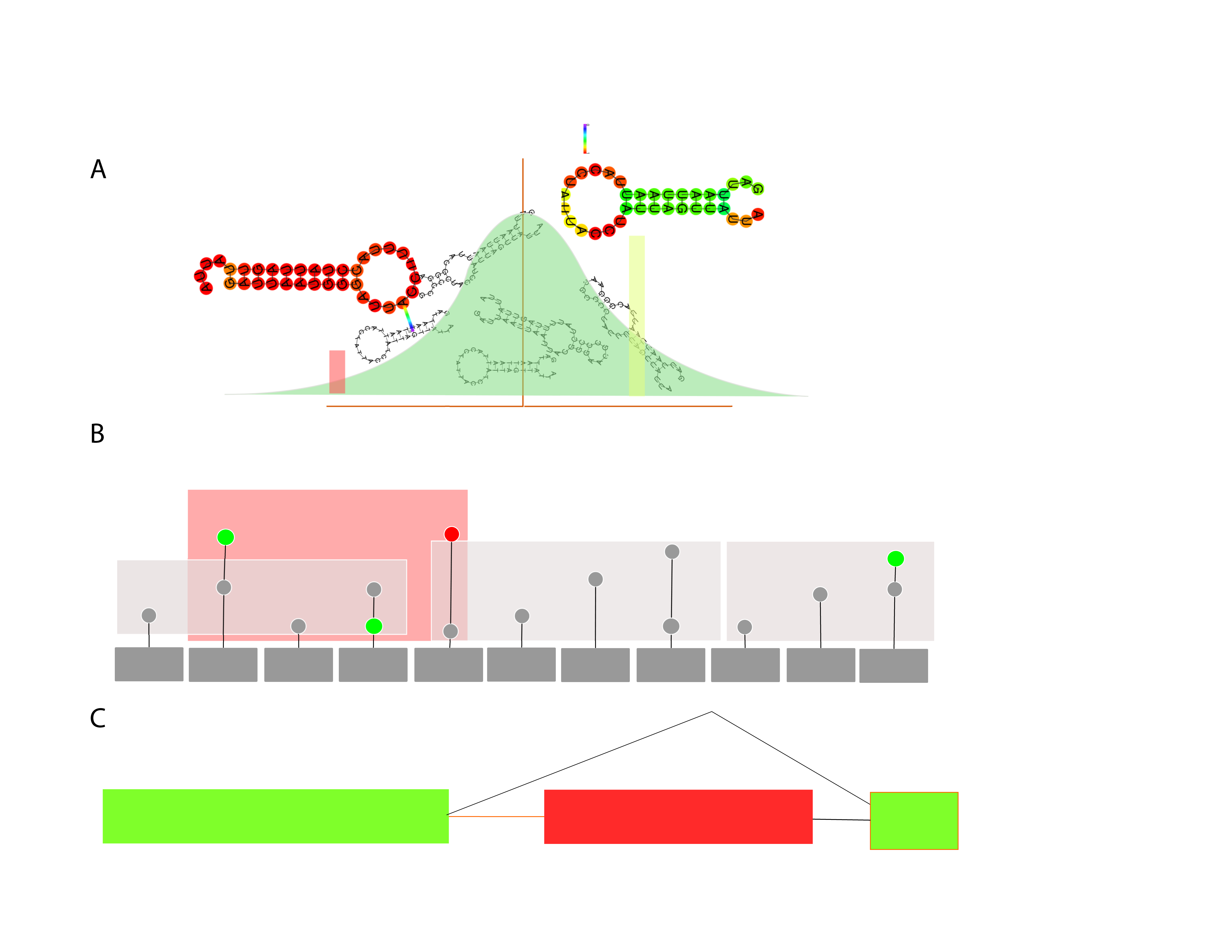

Supplement: Figure S4 — Synonymous variant risk prediction. Three models for the association between a synonymous SNVs and gene function. A, Shifts in signal to noise ratios between energies of a window of nucleotides that surround the SNV locus. The random background model is generated as sequences that have identical nucleotide composition except for a small interval that contains the SNV locus, thus measuring the contribution of the reference and polymorphic nucleotide to mRNA free energy, which is used as a proxy of mRNA stability. B, Codon usage frequencies correlate with ribosome latency and have been shown to affect, sometimes dramatically, protein elongation dynamics. Codons are clustered based on their position and usage frequencies, in both the reference and SNV-containing transcript. Changes in cluster centroids are given as a measure of local influences of codon frequency changes to global codon usage structure. C, Splicing site generation or disruption is measured as the change in predicted odds ratio of a maximum entropy splicing model. All synonymous SNVs were analyzed using these three criteria. (TIF) [file pgen.1002280.s004.tif]

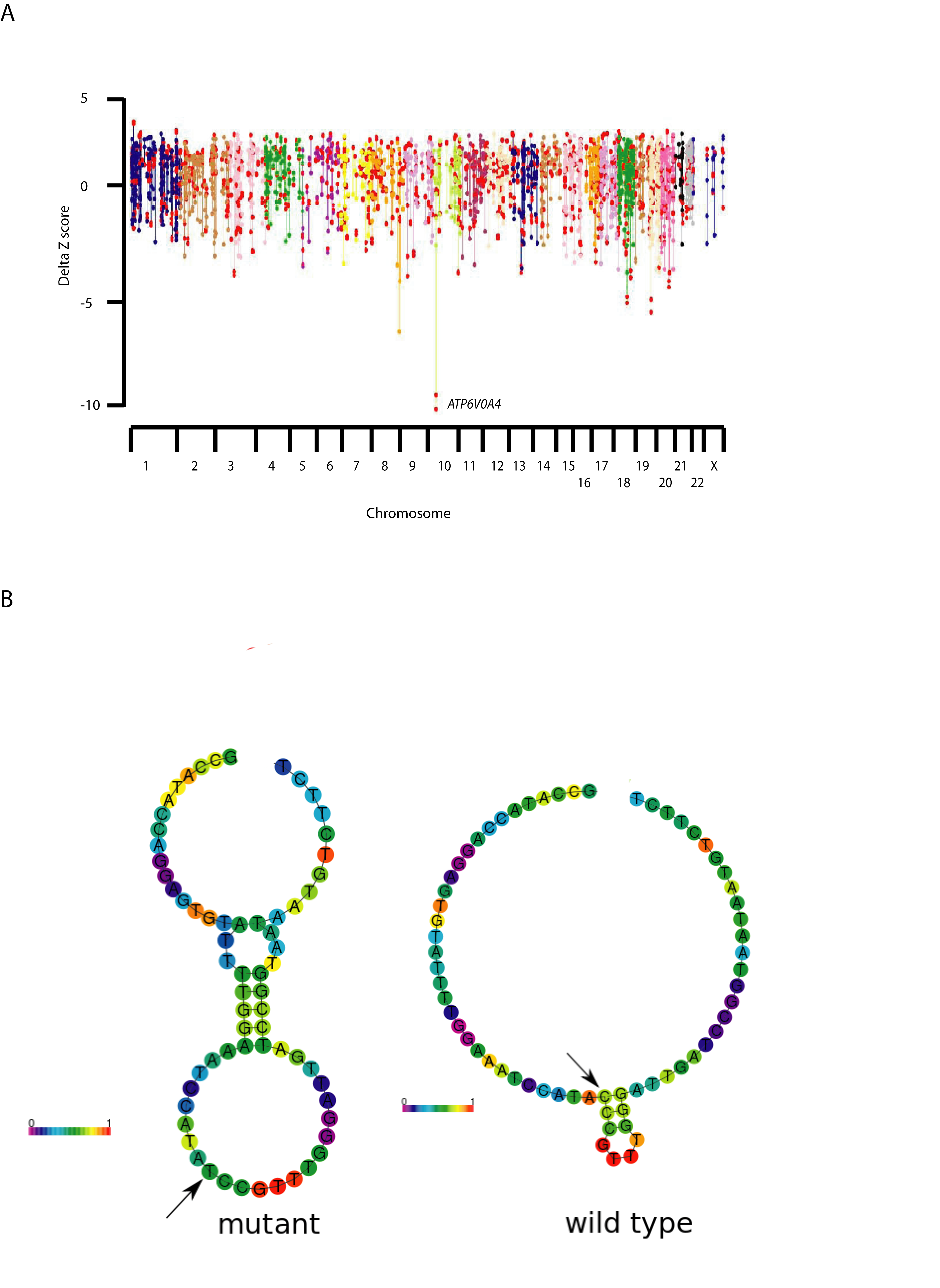

Supplement: Figure S5 — Synonymous SNV prediction identifies a putative loss of function variant in ATP6V0A4. A, A Z score method for predicting free energy change conferred by synonymous single nucleotide variants identified loci in the coding region of ATP6V0A4 associated with a significant change in mRNA free energy. B, Predicted change in mRNA secondary structure by C>T transition at rs74921348. (TIF) [file pgen.1002280.s005.tif]
