# Supplementary material for: Phased Whole-Genome Genetic Risk in a Family Quartet Using a Major Allele Reference Sequence
Source: PLoS Genet. 2011 Sep 15;7(9):e1002280. doi: 10.1371/journal.pgen.1002280 (PMC3174201; doi:10.1371/journal.pgen.1002280)
Supplement: Table S1 — Very rare (minor allele frequency <1%) disease risk alleles in the NCBI reference genome. (DOC) [file pgen.1002280.s006.doc]

Table S1. Very rare (minor allele frequency < 1%) disease risk alleles in the NCBI reference genome

| Disease/trait | dbSNP | symbol | Type | Risk allele | P value | Odds Ratio | Population with MAF < 1% |
| --- | --- | --- | --- | --- | --- | --- | --- |
| Blood pressure | 653178 | ATXN2 | intron | C | 3.00E-18 |  | ALL, CHB/JPT, YRI |
| Celiac disease | 653178 | ATXN2 | intron | C | 7.15E-21 | 1.20 | ALL, CHB/JPT, YRI |
| Celiac disease | 3184504 | SH2B3 | missense | T | 1.23E-12 | 1.21 | ALL, CHB/JPT, YRI |
| Coronary artery disease | 3184504 | SH2B3 | missense | T | 4.23E-11 | 1.14 | CHB/JPT, YRI |
| End-Stage renal disease | 2032487 | MYH9 | intron | C | 1.98E-15 | 2.61 | CHB/JPT |
| End-Stage renal disease | 4821480 | MYH9 | intron | G | 8.48E-17 | 2.29 | CHB/JPT |
| End-Stage renal disease | 4821481 | MYH9 | intron | C | 2.28E-16 | 2.62 | CHB/JPT |
| End-Stage renal disease | 5756152 | MYH9 | intron | A | 5.84E-11 | 2.82 | ALL, CHB/JPT |
| Focal segmental glomerulosclerosis | 4821481 | MYH9 | intron | C | 1.28E-09 |  | CHB/JPT |
| Ige levels | 1295685 | IL13 | UTR-3 | A | 2.00E-07 | 1.61 | YRI |
| Rheumatoid arthritis | 1217413 |  | intergenic | G | 4.00E-08 | 1.38 | YRI |
| Rheumatoid arthritis | 2476601 | PTPN22 | missense | A | 2.30E-98 | 1.96 | ALL, CHB/JPT, YRI |
| Systemic lupus erythematosus | 2476601 | PTPN22 | missense | A | 3.4E-12 | 1.57 | ALL, CHB/JPT, YRI |
| Type 1 diabetes | 2476601 | PTPN22 | missense | A | 2.11E-87 | 2.25 | ALL, CHB/JPT, YRI |
| Type 1 diabetes | 3184504 | SH2B3 | missense | T | 5.62E-31 | 1.24 | ALL, CHB/JPT, YRI |
| Type 2 diabetes | 5219 | KCNJ11 | missense | T | 6.7E-11 | 1.26 | YRI |
| Type 2 diabetes | 2970847 | PPARGC1A | cds-synon | T | 1.59e-18 | 2.10 | YRI |
| Vitiligo | 2476601 | PTPN22 | missense | A | 6.79E-09 | 1.82 | ALL, CHB/JPT, YRI |
