# Supplementary material for: Phased Whole-Genome Genetic Risk in a Family Quartet Using a Major Allele Reference Sequence
Source: PLoS Genet. 2011 Sep 15;7(9):e1002280. doi: 10.1371/journal.pgen.1002280 (PMC3174201; doi:10.1371/journal.pgen.1002280)
Supplement: Table S2 — BWA alignment efficiency using HG19 and the CEU major allele reference genomes. (DOC) [file pgen.1002280.s007.doc]

Tabl2 S2. BWA alignment efficiency using HG19 and the CEU major allele reference genomes

|  | HG19 reference | CEU reference |
| --- | --- | --- |
| Bases aligned – chr 22 | 13,757,281 | 13,758,089 |
| Bases aligned – chr 6 | 26,186,555 | 26,189,514 |
| Reads aligned – chr 22 | 3,740,839 | 3,741,223 |
| Reads aligned – chr6 | 4,503,067 | 4,503,444 |
