# Supplementary material for: Phased Whole-Genome Genetic Risk in a Family Quartet Using a Major Allele Reference Sequence
Source: PLoS Genet. 2011 Sep 15;7(9):e1002280. doi: 10.1371/journal.pgen.1002280 (PMC3174201; doi:10.1371/journal.pgen.1002280)
Supplement: Table S3 — Genotype changes for disease-associated variants using a major allele reference sequence. (DOC) [file pgen.1002280.s008.doc]

Table S3. Genotype changes for disease-risk associated variants using a major allele reference sequence

| Hg19 -> CEU change | Father | Mother | Son | Daughter |
| --- | --- | --- | --- | --- |
| Risk allele | 23 | 11 | 14 | 21 |
| Protective allele | 46 | 29 | 25 | 44 |
| No change | 11 | 63 | 68 | 14 |
| Missing risk allele | 48 | 25 | 21 | 49 |
| Unmatched allele^#^ | 2 | 2 | 2 | 2 |
