# Supplementary material for: Phased Whole-Genome Genetic Risk in a Family Quartet Using a Major Allele Reference Sequence
Source: PLoS Genet. 2011 Sep 15;7(9):e1002280. doi: 10.1371/journal.pgen.1002280 (PMC3174201; doi:10.1371/journal.pgen.1002280)
Supplement: Table S4 — Prioritization scheme for rare and novel single nucleotide variants. (DOC) [file pgen.1002280.s009.doc]

**Table S4.** Prioritization schema for rare and novel single nucleotide variants

| Scale | Definition | Level keyed | 1 | 2 | 3 | 4 | 5 | 6 | 7 | 8 | 9 | 10 |
| --- | --- | --- | --- | --- | --- | --- | --- | --- | --- | --- | --- | --- |
| Onset - earliest | earliest possible age of onset | phenotype | infantile | 1-10yo | 11-20yo | 21-50yo | >51yo |  |  |  |  |  |
| Onset - median | median age of onset | phenotype | infantile | 1-10yo | 11-20yo | 21-50yo | >51yo |  |  |  |  |  |
| Severity | worst case, untreated, accounting for morbidity and mortality | phenotype | rarely affects quality or quantity of life | rarely affects quality or quantity of life | mild impact on quality or quantity of life | mild impact on quality or quantity of life | moderate impact on quality or quantity of life | moderate impact on quality or quantity of life | severely limits either quality or quantity of life | severely limits either quality or quantity of life | severely limits both quality and quantity of life | severely limits both quality and quantity of life |
| Actionability | based on best treatment | phenotype | medical or surgical prevention available | medical or surgical prevention available | medical or surgical cure available | medical or surgical cure available | Condition is not curable but effective protection from most decrements in quality and quantity of life | Condition is not curable but effective protection from most decrements in quality and quantity of life | treatments are available, but they only minimally affect decrements in quality or quantity of life | treatments are available, but they only minimally affect decrements in quality or quantity of life | only palliative intervention available | only palliative intervention available |
| Lifetime risk | of any manifestation (primarily because that's what data is historically available) | phenotype | no clinical impact, children may be at risk | <20% | 21-30% | 31-40% | 41-50% | 51-60% | 61-70% | 71-80% | 81-90% | 91-100% |
| Pathogenicity | likelihood variant causes disease | variant | known benign, strong evidence | some evidence suggesting benign | Novel predicted benign | Novel predicted to be damaging by 1 program | Novel, predicted to be damaging by 2 or more programs | reported in one case | reported in multiple cases | segregates with disease in oen family | segregates with disease in 2 families | experimental evidence confirms pathogenicity |
