# Supplementary material for: Phased Whole-Genome Genetic Risk in a Family Quartet Using a Major Allele Reference Sequence
Source: PLoS Genet. 2011 Sep 15;7(9):e1002280. doi: 10.1371/journal.pgen.1002280 (PMC3174201; doi:10.1371/journal.pgen.1002280)
Supplement: Table S5 — Variants of potential significance in OMIM-curated disease associated genes. (DOC) [file pgen.1002280.s010.doc]

**Table S5**. Variants of potential significance in OMIM-curated disease associated genes

| Chromosome | Position | Reference | rsid | son paternal allele | son maternal allele | daughter paternal allele | daughter maternal allele | father allele 1 | father allel 2 | mother allele 1 | mother allele 2 | MAF | PP2 prediction | SIFT Prediction | Gene Symbol | OMIM diseases | mammal rate | mammal timespan | variant ancestral count |
| --- | --- | --- | --- | --- | --- | --- | --- | --- | --- | --- | --- | --- | --- | --- | --- | --- | --- | --- | --- |
| 19 | 577782 | G | novel | T | G | G | G | G | T | G | G | 0.003 | possibly damaging | DAMAGING | BSG | [Blood group, OK], 111380 (3) | 3.67 | 0.49 | 2 |
| 1 | 982722 | A | novel | G | A | G | A | G | A | A | A | 0.004 | benign | DAMAGING | AGRN | Myasthenia, limb-girdle, familial, 254300 (3) | 0.00 | 0.66 | 4 |
| 11 | 1775352 | C | novel | C | T | C | C | C | C | T | C | 0.003 | benign | TOLERATED | CTSD | Ceroid lipofuscinosis, neuronal (Batten type 2), 10, 610127 (3) | 1.11 | 0.65 | 5 |
| 16 | 2158570 | G | novel | G | G | G | G | G | G | G | A | 0.007 | probably damaging | DAMAGING | PKD1 | Polycystic kidney disease, adult type 1 | 0.84 | 0.86 | 0 |
| 4 | 3148570 | G | rs35892913 | A | G | A | G | A | G | G | G | 0.042 | benign | TOLERATED | HTT | Huntington disease, 143100 (3) | 0.48 | 0.76 | 5 |
| 4 | 3494956 | C | rs16844464 | C | C | C | C | C | C | C | T | 0.036 | probably damaging | TOLERATED | DOK7 | Fetal akinesia deformation sequence, 208150 (3) | 1.70 | 0.64 | 1 |
| 17 | 3563930 | G | novel | A | G | A | G | A | G | G | G | novel | possibly damaging | TOLERATED | CTNS | Cystinosis, atypical nephropathic (3) | 0.51 | 0.70 | 1 |
| 1 | 6528137 | G | novel | G | G | G | G | G | A | G | G | novel | benign | TOLERATED | PLEKHG5 | Spinal muscular atrophy, distal, autosomal recessive, 4, 611067 (3) | 1.69 | 0.85 | 2 |
| 9 | 6553445 | C | novel | C | T | C | C | C | C | T | C | novel | benign | TOLERATED | GLDC | Glycine encephalopathy, 605899 (3) | 1.79 | 0.81 | 12 |
| 12 | 7045178 | G | novel | G | G | G | G | G | A | G | G | 0.006 | unknown | TOLERATED | ATN1 | Dentatorubro-pallidoluysian atrophy, 125370 (3) | 0.60 | 0.61 | 3 |
| 19 | 11233886 | C | rs45508991 | T | C | C | C | T | C | C | C | 0.013 | benign | DAMAGING | LDLR | Hypercholesterolemia, familial, 143890 (3) | 0.66 | 0.55 | 0 |
| 16 | 14028081 | C | rs1799802 | T | C | T | C | T | C | C | C | 0.018 | probably damaging | DAMAGING | ERCC4 | XFE progeroid syndrome, 610965 (3) | 0.00 | 0.98 | 0 |
| 1 | 16378000 | A | rs6650119 | A | A | A | A | A | A | A | G | 0.012 | possibly damaging | DAMAGING | CLCNKB | Bartter syndrome, type 3, 607364 (3) | 1.00 | 0.72 | 1 |
| 1 | 19199448 | G | rs61757683 | G | T | G | G | G | G | T | G | 0.033 | probably damaging | DAMAGING | ALDH4A1 | Hyperprolinemia, type II, 239510 (3) | 0.00 | 0.77 | 0 |
| 11 | 22301090 | C | novel | C | C | G | C | C | G | C | C | novel | probably damaging | DAMAGING | ANO5 | Gnathodiaphyseal dysplasia, 166260 (3) | 0.00 | 0.92 | 0 |
| 16 | 23400257 | C | novel | T | C | T | C | T | C | C | C | novel | probably damaging | DAMAGING | COG7 | Congenital disorder of glycosylation, type IIe, 608779 (3) | 0.00 | 0.83 | 0 |
| 16 | 23409440 | G | rs16940094 | G | G | G | A | G | G | G | A | 0.030 | possibly damaging | TOLERATED | COG7 | Congenital disorder of glycosylation, type IIe, 608779 (3) | 0.78 | 0.92 | 2 |
| 16 | 28488943 | T | rs77595156 | T | C | T | C | T | T | C | T | 0.025 | probably damaging | TOLERATED | CLN3 | Ceroid-lipofuscinosis, neuronal-3, juvenile, 204200 (3) | 0.00 | 0.82 | 1 |
| 15 | 28517429 | G | novel | G | G | G | G | G | G | G | A | novel | probably damaging | DAMAGING | HERC2 | [Skin/hair/eye pigmentation 1, blond/brown hair], 227220 (3) | 0.00 | 0.90 | 1 |
| 18 | 29102187 | C | novel | C | C | C | T | C | C | C | T | novel | probably damaging | DAMAGING | DSG2 | Arrhythmogenic right ventricular dysplasia, familial, 10, 610193 (3) | 0.00 | 0.93 | 6 |
| 2 | 31560572 | G | rs45564939 | G | G | G | A | G | G | G | A | 0.007 | possibly damaging | DAMAGING | XDH | xanthinuria, type 1, 603707 | 0.00 | 0.94 | 1 |
| 22 | 36745146 | G | novel | G | G | G | A | G | G | G | A | novel | possibly damaging | DAMAGING | MYH9 | Deafness, autosomal dominant 17, 603622 (3) | 0.00 | 0.87 | 1 |
| 8 | 38162945 | G | novel | A | G | A | G | A | G | G | G | novel | probably damaging | TOLERATED | WHSC1L1 | Leukemia, acute myeloid, 601626 (3) | 0.00 | 0.91 | 0 |
| 22 | 40757509 | T | rs8192461 | T | T | T | C | T | T | T | C | 0.001 | probably damaging | DAMAGING | ADSL | Adenylosuccinase deficiency, 103050 (3) | 0.37 | 0.98 | 0 |
| 19 | 40903043 | C | rs117336941 | C | C | C | T | C | C | C | T | 0.017 | benign | TOLERATED | PRX | Charcot-Marie-Tooth disease, type 4F, 145900 (3) | 3.18 | 0.68 | 8 |
| 8 | 41582031 | G | rs61735313 | T | G | T | G | T | T | G | G | 0.025 | probably damaging | DAMAGING | ANK1 | Spherocytosis, type 1, 182900 (3) | 0.00 | 0.88 | 0 |
| 19 | 46274624 | G | novel | G | G | G | G | G | G | G | A | 0.006 | possibly damaging | DAMAGING | DMPK | Myotonic dystrophy, 160900 (3) | 0.53 | 0.68 | 1 |
| 1 | 46714242 | A | rs28363192 | A | G | A | A | A | A | G | A | 0.027 | benign | DAMAGING | RAD54L | Adenocarcinoma, colonic, somatic (3) | 0.39 | 0.93 | 0 |
| 12 | 51080364 | C | rs74751916 | T | C | C | C | T | C | C | C | 0.033 | possibly damaging | DAMAGING | DIP2B | Mental retardation, FRA12A type, 136630 (3) | 0.00 | 0.75 | 0 |
| 14 | 51372238 | T | rs34313873 | T | T | A | T | T | A | T | T | 0.008 | possibly damaging | DAMAGING | PYGL | Glycogen storage disease VI (3) | 0.00 | 0.98 | 0 |
| 5 | 52337983 | G | novel | A | G | A | G | A | G | G | G | novel | probably damaging | DAMAGING | ITGA2 | Glycoprotein Ia deficiency (1) (?) | 0.00 | 0.91 | 0 |
| 12 | 52710721 | G | rs2852464 | G | G | C | C | G | C | G | C | 0.022 | possibly damaging | DAMAGING | KRT83 | Monilethrix, 158000 (3) | 2.21 | 0.65 | 0 |
| 4 | 55127448 | G | rs36035373 | G | G | G | G | G | A | G | G | 0.007 | possibly damaging | DAMAGING | PDGFRA | Gastrointestinal stromal tumor, somatic, 606764 (3) | 0.00 | 0.95 | 0 |
| 20 | 56137130 | G | novel | G | G | G | G | G | T | G | G | novel | probably damaging | DAMAGING | PCK1 | Hypoglycemia due to PCK1 deficiency (1) | 0.00 | 0.90 | 0 |
| 17 | 56348106 | T | rs2759 | T | T | T | C | T | T | T | C | 0.018 | benign | TOLERATED | MPO | Myeloperoxidase deficiency, 254600 (3) | 0.00 | 0.71 | 1 |
| 17 | 61559041 | G | novel | G | G | A | G | G | A | G | G | novel | benign | TOLERATED | ACE | Renal tubular dysgenesis, 267430 (3) | 0.00 | 0.68 | 0 |
| 2 | 71762413 | G | rs61740288 | G | G | G | G | G | A | G | G | 0.025 | probably damaging | DAMAGING | DYSF | Miyoshi myopathy, 254130 (3) | 0.00 | 0.80 | 0 |
| 15 | 73617404 | G | novel | A | G | A | G | A | G | G | G | novel | unknown | DAMAGING | HCN4 | Brugada syndrome 8, 613123 (3) | 0.00 | 0.57 | 0 |
| 2 | 73679956 | C | rs28730854 | C | C | C | C | C | C | C | T | 0.017 | possibly damaging | DAMAGING | ALMS1 | Alstrom syndrome, 203800 (3) | 0.51 | 0.71 | 0 |
| 14 | 75513463 | A | rs17782839 | A | G | A | A | A | A | G | A | 0.013 | benign | TOLERATED | MLH3 | Colon cancer, hereditary nonpolyposis, type 7 (3) | 1.71 | 0.85 | 23 |
| 14 | 75515668 | T | rs28756981 | G | T | T | T | G | T | T | T | 0.013 | possibly damaging | TOLERATED | MLH3 | Colon cancer, hereditary nonpolyposis, type 7 (3) | 0.38 | 0.94 | 1 |
| 12 | 76740149 | G | rs35676114 | G | G | A | G | G | A | G | G | 0.033 | benign | TOLERATED | BBS10 | Bardet-Biedel syndrome 10, 209900 (3) | 1.74 | 0.83 | 9 |
| 17 | 78186067 | C | novel | C | C | C | G | C | C | C | G | novel | probably damaging | TOLERATED | SGSH | Sanfilippo syndrome, type A, 252900 (3) | 0.00 | 0.63 | 0 |
| 15 | 89868840 | C | novel | T | C | T | C | T | C | C | C | novel | probably damaging | TOLERATED | POLG | Alpers syndrome, 203700 (3) | 0.46 | 0.79 | 1 |
| 15 | 91326099 | C | rs11852361 | C | T | C | T | C | C | T | C | 0.043 | possibly damaging | DAMAGING | BLM | Bloom syndrome, 210900 (3) | 0.00 | 0.88 | 0 |
| 15 | 91354521 | G | rs7167216 | G | A | G | A | G | G | A | G | 0.025 | benign | TOLERATED | BLM | Bloom syndrome, 210900 (3) | 2.96 | 0.97 | 11 |
| 14 | 94844947 | C | rs28929474 | C | C | C | T | C | C | C | T | 0.017 | probably damaging | DAMAGING | SERPINA1 | Emphysema due to AAT deficiency, 613490 (3) | 0.92 | 0.78 | 1 |
| 8 | 95411644 | G | novel | G | G | G | G | G | T | G | G | novel | probably damaging | DAMAGING | RAD54B | Colon adenocarcinoma (3) | 0.39 | 0.93 | 0 |
| 12 | 102158764 | G | rs76889468 | G | G | A | G | G | A | G | G | 0.025 | possibly damaging | DAMAGING | GNPTAB | Mucolipidosis II alpha/beta, 252500 (3) | 0.37 | 0.97 | 0 |
| 11 | 103026168 | C | novel | C | C | C | A | C | C | C | A | novel | possibly damaging | DAMAGING | DYNC2H1 | Asphyxiating thoracic dystrophy 3, 613091 (3) | 2.02 | 0.89 | 1 |
| 9 | 104125056 | A | rs61755096 | A | A | G | A | A | G | A | A | 0.009 | benign | TOLERATED | BAAT | Hypercholanemia, familial, 607748 (3) | 6.10 | 0.83 | 9 |
| 11 | 108143456 | C | rs1800057 | C | G | C | C | C | C | G | C | 0.025 | probably damaging | DAMAGING | ATM | Ataxia-telangiectasia, 208900 (3) | 0.00 | 0.93 | 0 |
| 11 | 111635566 | C | rs1805076 | C | C | C | C | C | T | C | C | 0.006 | benign | TOLERATED | PPP2R1B | Lung cancer, 211980 (3) | 0.80 | 0.90 | 0 |
| 9 | 111692162 | G | novel | G | C | G | G | G | G | C | G | novel | probably damaging | TOLERATED | IKBKAP | Dysautonomia, familial, 223900 (3) | 0.00 | 0.95 | 0 |
| 5 | 112178795 | G | rs2229995 | G | G | G | A | G | G | G | A | 0.032 | benign | TOLERATED | APC | Adenoma, periampullary (3) | 0.00 | 0.95 | 4 |
| 10 | 112572458 | G | rs1417635 | C | C | C | C | C | C | C | C | 0.002 | benign | TOLERATED | RBM20 | Cardiomyopathy, dilated, 1DD, 613172 (3) | 2.59 | 0.98 | 31 |
| 11 | 123504870 | G | novel | G | A | G | G | G | G | A | G | novel | benign | TOLERATED | SCN3B | Brugada syndrome 7, 613120 (3) | 1.90 | 0.95 | 6 |
| 11 | 46747447 | G | rs62623459 | G | G | G | G | G | A | G | G | 0.001 | benign | TOLERATED | F2 | Hypoprothrombinemia (3 | 4.76 | 0.83 | 16 |
| 8 | 144995494 | C | rs6558407 | T | T | C | C | T | C | T | C | 0.043 | probably damaging | DAMAGING | PLEC1 | Epidermolysis bullosa simplex with pyloric atresia, 612138 (3) | 1.32 | 0.82 | 7 |
| 2 | 167108386 | G | novel | G | G | G | G | G | G | G | A | novel | possibly damaging | DAMAGING | SCN9A | Erythermalgia, primary, 133020 (3) | 0.76 | 0.95 | 0 |
| 2 | 170103472 | G | rs114842875 | G | G | G | G | G | G | G | A | 0.017 | probably damaging | DAMAGING | LRP2 | Donnai-Barrow syndrome, 222448 (3) | 0.77 | 0.94 | 1 |
| 3 | 187447701 | C | rs61752081 | C | C | C | C | C | A | C | C | 0.042 | benign | TOLERATED | BCL6 | Lymphoma, B-cell (2) | 1.25 | 0.87 | 0 |
| 1 | 197237571 | T | novel | T | C | T | T | T | T | C | T | novel | possibly damaging | DAMAGING | CRB1 | Leber congenital amaurosis 8 (3) | 0.00 | 0.87 | 0 |
| 2 | 203420712 | G | rs2228545 | G | A | G | A | G | G | A | G | 0.022 | benign | TOLERATED | BMPR2 | Pulmonary hypertension, familial primary, 178600 (3) | 0.38 | 0.96 | 3 |
| 2 | 228163453 | C | rs57611801 | A | C | C | C | C | A | A | C | 0.035 | unknown | TOLERATED | COL4A3 | Alport syndrome, autosomal recessive, 203780 (3) | 1.61 | 0.90 | 4 |
| 2 | 233659553 | C | rs2289912 | A | C | C | C | A | C | C | C | 0.017 | possibly damaging | TOLERATED | GIGYF2 | Parkinson disease 11, 607688 (3) | 0.88 | 0.82 | 0 |
