# Supplementary material for: Phased Whole-Genome Genetic Risk in a Family Quartet Using a Major Allele Reference Sequence
Source: PLoS Genet. 2011 Sep 15;7(9):e1002280. doi: 10.1371/journal.pgen.1002280 (PMC3174201; doi:10.1371/journal.pgen.1002280)
Supplement: Table S6 — Compound heterozygous and homozygous variants in Mendelian disease associated genes. (DOC) [file pgen.1002280.s011.doc]

**Table S6**. Compound heterozygous and homozygous variants in Mendelian disease associated genes

| Chromosome | Position | Reference | rsid | son paternal allele | son maternal allele | daughter paternal allele | daughter maternal allele | father allele 1 | father allel 2 | mother allele 1 | mother allele 2 | MAF | PP2 prediction | SIFT Prediction | Gene Symbol | OMIM diseases | mammal rate | mammal timespan | variant ancestral count |
| --- | --- | --- | --- | --- | --- | --- | --- | --- | --- | --- | --- | --- | --- | --- | --- | --- | --- | --- | --- |
| 8 | 144995494 | C | rs6558407 | T | T | C | C | T | C | T | C | 0.04 | probably damaging | DAMAGING | PLEC1 | Epidermolysis bullosa simplex with pyloric atresia, 612138 (3) | 1.32 | 0.82 | 7 |
| 12 | 52710721 | G | rs2852464 | G | G | C | C | G | C | G | C | 0.02 | possibly damaging | DAMAGING | KRT83 | Monilethrix, 158000 (3) | 2.21 | 0.65 | 0 |
| 8 | 17928811 | C | rs1071645 | C | C | T | T | C | T | C | T | 0.03 | benign | TOLERATED | ASAH1 | Farber lipogranulomatosis (3) | 3.06 | 0.82 | 11 |
| 15 | 91326099 | C | rs11852361 | C | T | C | T | C | C | T | C | 0.04 | possibly damaging | DAMAGING | BLM | Bloom syndrome, 210900 (3) | 0.00 | 0.88 | 0 |
| 15 | 91354521 | G | rs7167216 | G | A | G | A | G | G | A | G | 0.03 | benign | TOLERATED | BLM | Bloom syndrome, 210900 (3) | 2.96 | 0.97 | 11 |
| 2 | 74489318 | G | rs55651232 | G | A | G | A | G | G | A | G | 0.03 | possibly damaging | TOLERATED | SLC4A5 | Renal tubular acidosis, proximal, with ocular abnormalities, 604278 (3) | 0.00 | 0.71 | 0 |
| 2 | 74450058 | C | rs36081793 | T | C | C | C | T | C | C | C | 0.03 | possibly damaging | TOLERATED | SLC4A5 | Renal tubular acidosis, proximal, with ocular abnormalities, 604278 (3) | 0.38 | 0.96 | 1 |
| 14 | 75515668 | T | rs28756981 | G | T | T | T | G | T | T | T | 0.01 | possibly damaging | TOLERATED | MLH3 | Colon cancer, hereditary nonpolyposis, type 7 (3) | 0.38 | 0.94 | 1 |
| 14 | 75513463 | A | rs17782839 | A | G | A | A | A | A | G | A | 0.01 | benign | TOLERATED | MLH3 | Colon cancer, hereditary nonpolyposis, type 7 (3) | 1.71 | 0.85 | 23 |
| 16 | 23409440 | G | rs16940094 | G | G | G | A | G | G | G | A | 0.03 | possibly damaging | TOLERATED | COG7 | Congenital disorder of glycosylation, type IIe, 608779 (3) | 0.78 | 0.92 | 2 |
| 16 | 23400257 | C | novel | T | C | T | C | T | C | C | C | novel | probably damaging | DAMAGING | COG7 | Congenital disorder of glycosylation, type IIe, 608779 (3) | 0.00 | 0.83 | 0 |
