# Supplementary material for: Phased Whole-Genome Genetic Risk in a Family Quartet Using a Major Allele Reference Sequence
Source: PLoS Genet. 2011 Sep 15;7(9):e1002280. doi: 10.1371/journal.pgen.1002280 (PMC3174201; doi:10.1371/journal.pgen.1002280)
Supplement: Table S7 — Example pharmacogenomics annotation. (DOC) [file pgen.1002280.s012.doc]

**Table S7**. Example pharmacogenomics annotation

| **Proton Pump Inhibitors (such as Prilosec)** | |
| --- | --- |
|  |  |
| **Father** | **Probable typical response to proton pump inhibitors.** |
| Details: | CYP2C19*2/CYP2C19*17, one poor metabolizer allele and one ultra-rapid metabolizer allele: Response to proton pump inhibitors with this combination in alleles is not well-studied. The poor metabolizer allele associates with increased response to treatment for some indications, while the ultra-rapid metabolizer allele associates with decreased response. |
| **Mother** | **May not respond well to commonly prescribed dosages due to ultrarapid clearance of these drugs.** |
| Details: | CYP2C19*17 homozygous, ultra-rapid metabolizer: Is associated with increased proton pump inhibitor clearance and decreased efficacy. |
| **Sister** | **May not respond well to commonly prescribed dosages due to ultrarapid clearance of these drugs.** |
| Details: | CYP2C19*17 homozygous, ultra-rapid metabolizer: Is associated with increased proton pump inhibitor clearance and decreased efficacy. |
| **Brother** | **Probable typical response to proton pump inhibitors.** |
| Details: | CYP2C19*2/CYP2C19*17, one poor metabolizer allele and one ultra-rapid metabolizer allele: Response to proton pump inhibitors with this combination in alleles is not well-studied. The poor metabolizer allele associates with increased response to treatment for some indications, while the ultra-rapid metabolizer allele associates with decreased response. |
