# Supplementary material for: Phased Whole-Genome Genetic Risk in a Family Quartet Using a Major Allele Reference Sequence
Source: PLoS Genet. 2011 Sep 15;7(9):e1002280. doi: 10.1371/journal.pgen.1002280 (PMC3174201; doi:10.1371/journal.pgen.1002280)
Supplement: Table S8 — Variants associated with drug efficacy. (DOC) [file pgen.1002280.s013.doc]

**Table S8**. Variants associated with drug efficacy

| Key: Father, Mother, Brother, Sister = | | ◼¢◼¢ | Family members' genotypes as compared to other possible genotypes; not a population-based statistic | | | |
| --- | --- | --- | --- | --- | --- | --- |
| Gene Symbol | SNP | Drug(s) | Drug(s) More Likely to Work | Drug(s) Less Likely to Work | No PGx Action/ Phenotype Unknown | Confidence Level |
| *GRIK4* | rs1954787 | citalopram | ◼¢🞆 | ◼ |  | High |
| *LTA, TNF* | rs1800629 | adalimumab, etanercept, infliximab | ◼¢◼¢ |  |  | Medium |
| *VDR* | rs1544410 | alendronate, bisphosphonates, calcium, clodronate, etidronic acid, raloxifene |  | ◼¢◼¢ |  | Medium |
| *ABCB1* | rs2032583 | antidepressants | ◼ | ◼¢🞆 |  | Medium |
| *FKBP5* | rs3800373 | antidepressants |  | ◼¢◼¢ |  | Medium |
| *FKBP5* | rs1360780 | antidepressants |  | ◼¢◼¢ |  | Medium |
| *HTR2A* | rs7997012 | antidepressants, citalopram | ◼¢ | ◼¢ |  | Medium |
| *SERPINE1* | rs2227631 | antidepressants, citalopram, fluoxetine | 🞆 | ◼¢◼ |  | Medium |
| *NOS3* | rs2070744 | antihypertensives and diuretics in combination | ◼¢◼¢ |  |  | Medium |
| *PPARA* | rs4253778 | beta blocking agents | 🞆 | ◼¢◼ |  | Medium |
| *IL1B* | rs16944 | bisphosphonates, clodronate, etidronic acid, risedronate, tiludronate | nn | 🞆🞆 |  | Medium |
| *COMT* | rs165599 | bupropion | ◼¢◼¢ |  |  | Medium |
| *HTR3B* | rs1800497 | bupropion | ◼¢◼¢ |  |  | Medium |
| *GSTA1* | rs3957357 | cisplatin, cyclophosphamide |  | 🞆🞆 | nn | Medium |
| *ERCC2, KLC3* | rs13181 | cisplatin, fluorouracil, leucovorin, platinum compounds |  | ◼¢◼¢ |  | Medium |
| *SOD2* | rs4880 | cyclophosphamide | 🞆 | ◼¢◼ |  | Medium |
| *NOS3* | rs1799983 | cyclophosphamide, doxorubicin, fluorouracil, methotrexate | ◼¢◼¢ |  |  | Medium |
| *CYP1B1* | rs1056836 | docetaxel, paclitaxel, taxanes | ◼¢ | ¢◼ |  | Medium |
| *ABCB1* | rs1045642 | efavirenz, nelfinavir | ◼ | ◼¢🞆 |  | Medium |
| *CACNG2* | rs2284017 | lithium | ◼¢◼¢ |  |  | Medium |
| *SLC22A1* | rs622342 | metformin | ¢◼ | ◼¢ |  | Medium |
| *ABCG2* | rs13120400 | methotrexate | ◼¢◼¢ |  |  | Medium |
| *FPGS, ZYG11BL* | rs1544105 | methotrexate | nn | 🞆🞆 |  | Medium |
| *SLC19A1* | rs1051266 | methotrexate |  | ◼¢◼¢ |  | Medium |
| *ABCG2* | rs17731538 | methotrexate | ◼¢◼¢ |  |  | Medium |
| *MTHFR* | rs1801131 | methotrexate | ◼¢◼¢ |  |  | Medium |
| *ADM, SBF2* | rs11042725 | paroxetine | ◼¢◼¢ |  |  | Medium |
| *intergenic* | rs1695 | platinum compounds |  | ◼¢◼¢ |  | Medium |
| *KIF6* | rs20455 | pravastatin | ◼¢◼¢ |  |  | Medium |
| *FCGR3A* | rs396991 | rituximab | ◼¢ | ¢◼ |  | Medium |
| *ABCG2* | rs2231142 | rosuvastatin |  | ◼¢◼¢ |  | Medium |
| *ABCG2* | rs2231142 | rosuvastatin |  | ◼¢◼¢ |  | Medium |
| *CRHR2* | rs7793837 | salbutamol, selective beta-2-adrenoreceptor agonists |  | ◼¢◼¢ |  | Medium |
| *ACE* | rs4343 | sildenafil |  | nn | 🞆🞆 | Medium |
| *MBOAT5, GNB3, USP5* | rs5443 | sildenafil |  | nn | 🞆🞆 | Medium |
| *MBOAT5, GNB3, USP5* | rs5443 | statins | ◼¢ | ◼¢ |  | Medium |
| *TCF7L2* | rs12255372 | sulfonamides, urea derivatives | ◼ | ◼¢🞆 |  | Medium |
| *MBOAT5, GNB3, USP5* | rs5443 | sumatriptan | ◼¢ | ◼¢ |  | Medium |
| *intergenic* | rs10811661 | troglitazone | ◼¢◼¢ |  |  | Medium |
| *HTR1A* | rs6295 | antidepressants | nn¢ | 🞆 |  | Low |
| *ABCB1* | rs2235015 | antidepressants | ◼ | ◼¢🞆 |  | Low |
| *TPH2* | rs10879346 | antidepressants, mirtazapine, venlafaxine | ◼¢ | ¢◼ |  | Low |
| *LDLR* | rs688 | atenolol | nn | 🞆🞆 |  | Low |
| *FDPS, PKLR* | rs2297480 | bisphosphonates | ◼¢◼¢ |  |  | Low |
| *CCND1* | rs9344 | cetuximab | ◼¢◼¢ |  |  | Low |
| *ERCC2, KLC3* | rs13181 | cisplatin |  | ◼¢◼¢ |  | Low |
| *VDR* | rs731236 | clodronate |  | ◼¢◼¢ |  | Low |
| *DTNBP1* | rs742105 | clozapine | ◼¢◼¢ |  |  | Low |
| *ABCB1* | rs2032582 | cyclosporine | ◼¢ | ◼ |  | Low |
| *CDA* | rs532545 | cytarabine | nn | 🞆🞆 |  | Low |
| *CHST3* | rs4148943 | docetaxel, thalidomide | ¢◼ | ◼¢ |  | Low |
| *CHST3* | rs4148945 | docetaxel, thalidomide | ¢◼ | ◼¢ |  | Low |
| *CHST3* | rs4148947 | docetaxel, thalidomide | ¢◼ | ◼¢ |  | Low |
| *CHST3* | rs4148950 | docetaxel, thalidomide | ◼¢◼¢ |  |  | Low |
| *CHST3* | rs1871450 | docetaxel, thalidomide | ◼¢◼¢ |  |  | Low |
| *CHST3* | rs730720 | docetaxel, thalidomide | ¢◼ | ◼¢ |  | Low |
| *CHST3* | rs12418 | docetaxel, thalidomide | ◼¢◼¢ |  |  | Low |
| *PPARD* | rs2016520 | docetaxel, thalidomide | ◼¢◼¢ |  |  | Low |
| *PPARD* | rs2016520 | docetaxel, thalidomide | ◼¢◼¢ |  |  | Low |
| *PPARD* | rs6922548 | docetaxel, thalidomide |  | nn | 🞆🞆 | Low |
| *SULT1C4* | rs1402467 | docetaxel, thalidomide |  | nn | 🞆🞆 | Low |
| *PPARD* | rs1883322 | docetaxel, thalidomide | nn |  | 🞆🞆 | Low |
| *PPARD* | rs7769719 | docetaxel, thalidomide | nn |  | 🞆🞆 | Low |
| *PTGS2* | rs20417 | ibuprofen | 🞆🞆 | nn |  | Low |
| *APOB* | rs1367117 | irbesartan | ◼¢◼ | 🞆 |  | Low |
| *PKD1L3, DHODH* | rs3213422 | leflunomide |  | 🞆 | nn¢ | Low |
| *CYP19A1* | rs4646 | letrozole | ◼¢🞆 | ◼ |  | Low |
| *TLR3* | rs3775291 | measles vaccines | ◼¢◼¢ |  |  | Low |
| *TLR3* | rs3775291 | measles vaccines | ◼¢◼¢ |  |  | Low |
| *ARID5B* | rs10821936 | methotrexate | ◼¢ | ¢◼ |  | Low |
| *ATIC* | rs2372536 | methotrexate |  | ◼¢◼¢ |  | Low |
| *TPH2* | rs1487278 | mirtazapine, venlafaxine | ◼¢ | ¢◼ |  | Low |
| *ABCC1* | rs119774 | montelukast |  | ◼¢◼¢ |  | Low |
| *ALOX5* | rs2115819 | montelukast | ◼ | ¢◼¢ |  | Low |
| *LTC4S* | rs730012 | montelukast | ◼¢◼¢ |  |  | Low |
| *RMST* | rs2660845 | montelukast | 🞆 | nn¢ |  | Low |
| *SLCO2B1* | rs12422149 | montelukast |  | ◼¢◼ | 🞆 | Low |
| *ABCC1* | rs119774 | montelukast |  | ◼¢◼¢ |  | Low |
| *IMPDH1* | rs2278294 | mycophenolate mofetil | ¢◼ | ◼¢ |  | Low |
| *IMPDH1* | rs2278293 | mycophenolate mofetil | ¢◼¢ | ◼ |  | Low |
| *DRD3* | rs6280 | olanzapine |  | ◼¢◼¢ |  | Low |
| *HTR2A* | rs6313 | olanzapine, risperidone | 🞆 | nn¢ |  | Low |
| *ABCB1* | rs2032582 | paclitaxel |  | 🞆🞆 | nn | Low |
| *SLCO1B1* | rs4149015 | pravastatin | nn | 🞆🞆 |  | Low |
| *ABCB1* | rs1045642 | prednisone, tacrolimus | ◼¢◼ | 🞆 |  | Low |
| *SLCO1B1* | rs2306283 | repaglinide | nn |  | 🞆🞆 | Low |
| *ABCB1* | rs1128503 | risperidone | ◼¢◼ | 🞆 |  | Low |
| *COMT* | rs165599 | risperidone |  | ◼¢◼¢ |  | Low |
| *DRD3* | rs6280 | risperidone |  | ◼¢◼¢ |  | Low |
| *GRM3* | rs724226 | risperidone | 🞆🞆 |  | nn | Low |
| *HTR2A* | rs6311 | risperidone | nn¢ | 🞆 |  | Low |
| *RGS4* | rs951439 | risperidone | 🞆 | nn¢ |  | Low |
| *RGS4* | rs2842030 | risperidone | 🞆🞆 | nn |  | Low |
| *PTGS2* | rs20417 | rofecoxib | nn | 🞆🞆 |  | Low |
| *ADRB2* | rs1042713 | salmeterol | ◼¢◼¢ |  |  | Low |
| *ACE* | rs4343 | spironolactone | ¢◼¢ | ◼ |  | Low |
| *CSTP1* | rs6138150 | tumor necrosis factor alpha (TNF-alpha) inhibitors |  | ◼¢◼¢ |  | Low |
| *GBP6* | rs928655 | tumor necrosis factor alpha (TNF-alpha) inhibitors | nn¢ | 🞆 |  | Low |
| *MOBKL2B* | rs868856 | tumor necrosis factor alpha (TNF-alpha) inhibitors | ◼¢◼¢ |  |  | Low |
| *PON1* | rs854555 | tumor necrosis factor alpha (TNF-alpha) inhibitors | ◼¢◼¢ |  |  | Low |
| *PPP1R9A* | rs854547 | tumor necrosis factor alpha (TNF-alpha) inhibitors |  | ◼¢◼¢ |  | Low |
| *PPP1R9A, PON1* | rs854548 | tumor necrosis factor alpha (TNF-alpha) inhibitors | ◼¢◼¢ |  |  | Low |
| *intergenic* | rs983332 | tumor necrosis factor alpha (TNF-alpha) inhibitors | ◼ | ¢◼¢ |  | Low |
| *intergenic* | rs3849942 | tumor necrosis factor alpha (TNF-alpha) inhibitors | ◼¢◼¢ |  |  | Low |
| *intergenic* | rs6028945 | tumor necrosis factor alpha (TNF-alpha) inhibitors | 🞆 | ◼¢◼ |  | Low |
| *intergenic* | rs6071980 | tumor necrosis factor alpha (TNF-alpha) inhibitors | 🞆 | ◼¢◼ |  | Low |
| *intergenic* | rs437943 | tumor necrosis factor alpha (TNF-alpha) inhibitors |  | ◼¢◼¢ |  | Low |
| *C9orf72* | rs774359 | tumor necrosis factor alpha (TNF-alpha) inhibitors | ◼¢◼¢ |  |  | Low |
| *MOBKL2B* | rs2814707 | tumor necrosis factor alpha (TNF-alpha) inhibitors | ◼¢◼¢ |  |  | Low |
| *C9orf72* | rs774359 | tumor necrosis factor alpha (TNF-alpha) inhibitors | ◼¢◼¢ |  |  | Low |
| *LTA, TNF* | rs1800629 | adalimumab, etanercept, infliximab | ◼¢◼¢ |  |  | Medium |
| *VDR* | rs1544410 | alendronate, bisphosphonates, calcium, clodronate, etidronic acid, raloxifene |  | ◼¢◼¢ |  | Medium |
| *ABCB1* | rs2032583 | antidepressants | ◼ | ◼¢🞆 |  | Medium |
| *FKBP5* | rs3800373 | antidepressants |  | ◼¢◼¢ |  | Medium |
| *FKBP5* | rs1360780 | antidepressants |  | ◼¢◼¢ |  | Medium |
| *HTR2A* | rs7997012 | antidepressants, citalopram | ◼¢ | ◼¢ |  | Medium |
| *SERPINE1* | rs2227631 | antidepressants, citalopram, fluoxetine | 🞆 | ◼¢◼ |  | Medium |
| *NOS3* | rs2070744 | antihypertensives and diuretics in combination | ◼¢◼¢ |  |  | Medium |
| *PPARA* | rs4253778 | beta blocking agents | 🞆 | ◼¢◼ |  | Medium |
| *IL1B* | rs16944 | bisphosphonates, clodronate, etidronic acid, risedronate, tiludronate | nn | 🞆🞆 |  | Medium |
| *ARVCF, COMT* | rs165599 | bupropion | ◼¢◼¢ |  |  | Medium |
| *HTR3B* | rs1800497 | bupropion | ◼¢◼¢ |  |  | Medium |
| *GSTA1* | rs3957357 | cisplatin, cyclophosphamide |  | 🞆🞆 | nn | Medium |
| *ERCC2, KLC3* | rs13181 | cisplatin, fluorouracil, leucovorin, platinum compounds |  | ◼¢◼¢ |  | Medium |
| *SOD2* | rs4880 | cyclophosphamide | 🞆 | ◼¢◼ |  | Medium |
| *NOS3* | rs1799983 | cyclophosphamide, doxorubicin, fluorouracil, methotrexate | ◼¢◼¢ |  |  | Medium |
| *CYP1B1* | rs1056836 | docetaxel, paclitaxel, taxanes | ◼¢ | ¢◼ |  | Medium |
| *ABCB1* | rs1045642 | efavirenz, nelfinavir | ◼ | ◼¢🞆 |  | Medium |
| *CACNG2* | rs2284017 | lithium | ◼¢◼¢ |  |  | Medium |
| *SLC22A1* | rs622342 | metformin | ¢◼ | ◼¢ |  | Medium |
| *ABCG2* | rs13120400 | methotrexate | ◼¢◼¢ |  |  | Medium |
| *FPGS, ZYG11BL* | rs1544105 | methotrexate | nn | 🞆🞆 |  | Medium |
| *SLC19A1* | rs1051266 | methotrexate |  | ◼¢◼¢ |  | Medium |
| *ABCG2* | rs17731538 | methotrexate | ◼¢◼¢ |  |  | Medium |
| *MTHFR* | rs1801131 | methotrexate | ◼¢◼¢ |  |  | Medium |
| *ADM, SBF2* | rs11042725 | paroxetine | ◼¢◼¢ |  |  | Medium |
| *intergenic* | rs1695 | platinum compounds |  | ◼¢◼¢ |  | Medium |
| *KIF6* | rs20455 | pravastatin | ◼¢◼¢ |  |  | Medium |
| *FCGR3A* | rs396991 | rituximab | ◼¢ | ¢◼ |  | Medium |
| *ABCG2* | rs2231142 | rosuvastatin |  | ◼¢◼¢ |  | Medium |
| *ABCG2* | rs2231142 | rosuvastatin |  | ◼¢◼¢ |  | Medium |
| *CRHR2* | rs7793837 | salbutamol, selective beta-2-adrenoreceptor agonists |  | ◼¢◼¢ |  | Medium |
| *ACE* | rs4343 | sildenafil |  | nn | 🞆🞆 | Medium |
| *MBOAT5, GNB3, USP5* | rs5443 | sildenafil |  | nn | 🞆🞆 | Medium |
| *MBOAT5, GNB3, USP5* | rs5443 | statins | ◼¢ | ◼¢ |  | Medium |
| *TCF7L2* | rs12255372 | sulfonamides, urea derivatives | ◼ | ◼¢🞆 |  | Medium |
| *MBOAT5, GNB3, USP5* | rs5443 | sumatriptan | ◼¢ | ◼¢ |  | Medium |
| intergenic | rs10811661 | troglitazone | ◼¢◼¢ |  |  | Medium |
