# Supplementary material for: Phased Whole-Genome Genetic Risk in a Family Quartet Using a Major Allele Reference Sequence
Source: PLoS Genet. 2011 Sep 15;7(9):e1002280. doi: 10.1371/journal.pgen.1002280 (PMC3174201; doi:10.1371/journal.pgen.1002280)
Supplement: Table S9 — Variants associated with adverse drug response. (DOC) [file pgen.1002280.s014.doc]

**Table S9.** Variants associated with adverse drug response

| Key: Father, Mother, Brother, Sister = | | ◼¢◼¢ | Family members' genotypes as compared to other possible genotypes; not a population-based statistic | | | |
| --- | --- | --- | --- | --- | --- | --- |
| Gene Symbol | SNP Location | Drug(s) | Drug(s) More Likely to Cause Side Effect | Drug(s) Less Likely to Cause Side Effect | No PGx Action/ Phenotype Unknown | Confidence Level |
| TPMT | rs1800460 | purine analogues |  | ◼¢◼¢ |  | High |
| HTR3B | rs1800497 | antipsychotics | ◼¢◼¢ |  |  | Medium |
| HTR2C | rs1414334 | antipsychotics, clozapine, risperidone | ◼¢ | ¢◼ |  | Medium |
| ARVCF, COMT | rs9332377 | cisplatin |  | ◼¢◼¢ |  | Medium |
| COMT | rs4646316 | cisplatin | ◼¢◼ | 🞆 |  | Medium |
| ARVCF, COMT | rs9332377 | cisplatin |  | ◼¢◼¢ |  | Medium |
| NHLRC1, TPMT | rs1142345 | cisplatin |  | ◼¢ | ◼¢ | Medium |
| TPMT | rs1800460 | cisplatin |  | ◼¢ | ◼¢ | Medium |
| XRCC1 | rs25487 | cisplatin, cyclophosphamide | nn | 🞆🞆 |  | Medium |
| FAM119A, CREB1 | rs7569963 | citalopram | ◼ | ◼ | 🞆🞆 | Medium |
| ABCC1 | rs45511401 | doxorubicin | nn¢ | 🞆 |  | Medium |
| ABCC2 | rs17222723 | doxorubicin | nn | 🞆🞆 |  | Medium |
| ABCC2 | rs8187710 | doxorubicin | nn | 🞆🞆 |  | Medium |
| CYBA | rs4673 | doxorubicin | 🞆 | nn¢ |  | Medium |
| NCF4 | rs1883112 | doxorubicin |  | ◼¢◼¢ |  | Medium |
| RAC2 | rs13058338 | doxorubicin | 🞆🞆 | nn |  | Medium |
| ABCB1 | rs1045642 | efavirenz, nelfinavir | ◼ | ◼¢🞆 |  | Medium |
| CYP1A2 | rs762551 | leflunomide |  | ◼¢◼¢ |  | Medium |
| PICK1, ENTHD1 | rs2076369 | methamphetamine |  | 🞆 | nn¢ | Medium |
| ADORA2A | rs2298383 | methotrexate | ◼¢◼ | 🞆 |  | Medium |
| ADORA2A | rs3761422 | methotrexate | ◼¢◼¢ |  |  | Medium |
| ADORA2A | rs2267076 | methotrexate | ◼¢◼¢ |  |  | Medium |
| ADORA2A | rs2236624 | methotrexate | ◼¢◼¢ |  |  | Medium |
| ADORA2A, CYTSA | rs5760410 | methotrexate | ◼¢◼¢ |  |  | Medium |
| SLC19A1 | rs1051266 | methotrexate |  | ◼¢◼¢ |  | Medium |
| ABCC1 | rs246240 | methotrexate | ◼¢◼¢ |  |  | Medium |
| REN, ETNK2 | rs2368564 | muraglitazar | ◼ | ◼¢🞆 |  | Medium |
| EDN1 | rs5370 | muraglitazar |  | ◼¢◼¢ |  | Medium |
| CHRNA4 | rs2236196 | nicotine | 🞆🞆 | nn |  | Medium |
| CHRNA4 | rs1044396 | nicotine | 🞆🞆 | nn |  | Medium |
| intergenic | rs10115383 | nicotine |  | ◼¢◼¢ |  | Medium |
| MTHFR | rs1801131 | nitrous oxide |  | ◼¢◼¢ |  | Medium |
| HTR2C | rs518147 | olanzapine | ¢◼ | ◼ | 🞆 | Medium |
| EPHX1 | rs1051740 | phenytoin | 🞆🞆 |  | nn | Medium |
| EPHX1 | rs1051740 | phenytoin | 🞆🞆 |  | nn | Medium |
| EPHX1 | rs2234922 | phenytoin |  | 🞆🞆 | nn | Medium |
| intergenic | rs1695 | platinum compounds | ◼¢◼¢ |  |  | Medium |
| SLCO1B1 | rs4149056 | pravastatin |  | ◼¢◼¢ |  | Medium |
| APOA4, APOA1, APOC3 | rs5128 | ritonavir | nn¢ | 🞆 |  | Medium |
| APOA4, APOC3 | rs2854117 | ritonavir | nn¢ | 🞆 |  | Medium |
| APOA4, APOC3 | rs2854116 | ritonavir | nn¢ | 🞆 |  | Medium |
| BDKRB2 | rs8012552 | ace inhibitors, plain |  | ◼¢◼¢ |  | Low |
| PTGER3 | rs11209716 | ace inhibitors, plain |  | nn¢ | 🞆 | Low |
| CYP2C8 | rs10509681 | amodiaquine |  | ◼¢◼¢ |  | Low |
| CYP2C8 | rs10509681 | amodiaquine |  | ◼¢◼¢ |  | Low |
| DSCR9, CBR3 | rs1056892 | anthracyclines and related substances | ◼¢◼¢ |  |  | Low |
| HTR3B | rs1800497 | antipsychotics |  | ◼¢◼¢ |  | Low |
| HTR3B | rs2276307 | atorvastatin, pravastatin, simvastatin | nn¢ | 🞆 |  | Low |
| HTR7 | rs1935349 | atorvastatin, pravastatin, simvastatin | nn | 🞆🞆 |  | Low |
| NHLRC1, TPMT | rs1142345 | azathioprine, mercaptopurine, purine analogues |  | ◼¢◼¢ |  | Low |
| HSPA1A, C6orf21, HSPA1L | rs2227956 | carbamazepine | ¢◼ | ◼¢ |  | Low |
| LTA, TNF | rs1800629 | carbamazepine |  | ◼¢◼¢ |  | Low |
| LRP2 | rs2075252 | cisplatin | 🞆 | nn¢ |  | Low |
| SLC22A2 | rs316019 | cisplatin | ◼¢◼¢ |  |  | Low |
| XPC, TMEM43 | rs2228001 | cisplatin | ◼¢◼¢ |  |  | Low |
| ERCC1 | rs11615 | cisplatin, cyclophosphamide | ◼¢◼ | 🞆 |  | Low |
| ERCC1, CD3EAP, PPP1R13L | rs3212986 | cisplatin, cyclophosphamide | ◼¢◼ | 🞆 |  | Low |
| DRD2 | rs6277 | clozapine, olanzapine | ◼¢◼ | 🞆 |  | Low |
| HTR3B | rs1800497 | clozapine, olanzapine |  | ◼¢◼¢ |  | Low |
| CYP3A4 | rs2740574 | cyclophosphamide |  | ◼¢◼¢ |  | Low |
| CDA | rs532545 | cytarabine | 🞆🞆 | nn |  | Low |
| ABCC6 | rs2238472 | docetaxel, thalidomide | ◼¢◼¢ |  |  | Low |
| CHST3 | rs4148945 | docetaxel, thalidomide | ¢◼ | ◼¢ |  | Low |
| CHST3 | rs4148950 | docetaxel, thalidomide | ◼¢◼¢ |  |  | Low |
| CYP4B1 | rs4646487 | docetaxel, thalidomide | 🞆🞆 | nn |  | Low |
| NAT2 | rs1799931 | docetaxel, thalidomide | ◼¢◼¢ |  |  | Low |
| RPL13, SPG7 | rs12960 | docetaxel, thalidomide | ¢◼¢ | ◼ |  | Low |
| SLC10A2 | rs2301159 | docetaxel, thalidomide | 🞆🞆 | nn |  | Low |
| SPG7 | rs2292954 | docetaxel, thalidomide | ¢◼¢ | ◼ |  | Low |
| SLC22A16 | rs714368 | doxorubicin, doxorubicinol |  | ◼¢◼¢ |  | Low |
| CELF4 | rs4799915 | iloperidone | ◼¢ | ¢◼ |  | Low |
| CERKL | rs993648 | iloperidone | ◼ | ¢◼¢ |  | Low |
| NRG3 | rs4933824 | iloperidone | ◼¢◼¢ |  |  | Low |
| NUBPL | rs7142881 | iloperidone | ¢◼¢ | ◼ |  | Low |
| ABCB1 | rs1045642 | nortriptyline | ◼ | ◼¢🞆 |  | Low |
| HTR2A | rs7997012 | olanzapine |  | ◼¢◼¢ |  | Low |
| CYP2C8 | rs1113129 | paclitaxel | ◼¢ |  | ¢◼ | Low |
| CYP3A5 | rs776746 | paclitaxel |  | ◼¢◼¢ |  | Low |
| CYP2C8 | rs1934951 | pamidronate, zoledronate | ◼¢◼¢ |  |  | Low |
| AGTR1 | rs5182 | perindopril | ◼¢◼¢ |  |  | Low |
| DRD3 | rs167771 | risperidone |  | ◼¢◼¢ |  | Low |
| LEP | rs7799039 | risperidone | ◼¢◼¢ |  |  | Low |
| HTR3B | rs1800497 | risperidone |  | ◼¢◼¢ |  | Low |
| DRD2 | rs1799978 | risperidone |  | ◼¢◼¢ |  | Low |
| ESR2 | rs4986938 | tamoxifen |  | 🞆🞆 | nn | Low |
| ESR1 | rs9340799 | tamoxifen |  | 🞆🞆 | nn | Low |
| NOS1AP | rs10918594 | verapamil | ◼ |  | ◼¢🞆 | Low |
| NOS1AP | rs10494366 | verapamil | ◼ | 🞆 | ◼¢ | Low |
