# Supplementary material for: Phased Whole-Genome Genetic Risk in a Family Quartet Using a Major Allele Reference Sequence
Source: PLoS Genet. 2011 Sep 15;7(9):e1002280. doi: 10.1371/journal.pgen.1002280 (PMC3174201; doi:10.1371/journal.pgen.1002280)
Supplement: Table S10 — Variants associated with drug dosing. (DOC) [file pgen.1002280.s015.doc]

**Table S10.** Variants associated with drug dosing

| Key: Father, Mother, Brother, Sister = | | n¢n¢ | Family members' genotypes as compared to other possible genotypes; not a population-based statistic | | | | | |
| --- | --- | --- | --- | --- | --- | --- | --- | --- |
| Gene Symbol | SNP Location | Drug(s) | Drug Dose(s) Easy to Predict | Drug Dose(s) Difficult to Predict | Drug Dose(s) Above Average | Drug Dose(s) Below Average | No PGx Action/ Phenotype Unknown | Confidence Level |
| CYP3A5 | rs776746 | cyclosporine |  |  |  | ◼¢◼¢ |  | High |
| CYP4F2 | rs2108622 | acenocoumarol, warfarin |  |  |  | ◼¢◼¢ |  | Medium |
| CYP4F2 | rs2108622 | acenocoumarol, warfarin |  |  |  | ◼¢◼¢ |  | Medium |
| SCN1A | rs3812718 | carbamazepine |  |  | ¢◼¢ |  | ◼ | Medium |
| NALCN | rs7992226 | methotrexate |  | ◼¢◼¢ |  |  |  | Medium |
| COMT | rs4680 | morphine |  |  | ◼¢◼¢ |  |  | Medium |
| OPRM1 | rs1799971 | morphine |  |  |  | ◼¢◼¢ |  | Medium |
| SCN1A | rs3812718 | phenytoin |  |  | ¢◼¢ |  | ◼ | Medium |
| EPHX1 | rs2292566 | warfarin |  |  | ◼¢◼¢ |  |  | Medium |
| STX4 | rs10871454 | warfarin |  |  |  | ◼¢◼¢ |  | Medium |
| VKORC1 | rs8050894 | warfarin |  |  |  | ◼¢◼¢ |  | Medium |
| CYP3A4 | rs2740574 | docetaxel | ◼¢◼¢ |  |  |  |  | Low |
| ABCB1 | rs1045642 | fexofenadine | ◼¢🞆 |  | ◼ |  |  | Low |
| CYP3A4 | rs2740574 | indinavir | ◼¢◼¢ |  |  |  |  | Low |
| SLCO1B1 | rs4149056 | methotrexate | ◼¢◼¢ |  |  |  |  | Low |
| SLCO1B1 | rs2306283 | pravastatin |  | ◼¢◼¢ |  |  |  | Low |
| CYP3A4 | rs2740574 | tacrolimus | ◼¢◼¢ |  |  |  |  | Low |
| ABCC4 | rs1751034 | tenofovir |  | ◼¢◼¢ |  |  |  | Low |
| GSTP1 | rs1138272 | thiotepa |  | ◼¢◼¢ |  |  |  | Low |
