# Supplementary material for: Phased Whole-Genome Genetic Risk in a Family Quartet Using a Major Allele Reference Sequence
Source: PLoS Genet. 2011 Sep 15;7(9):e1002280. doi: 10.1371/journal.pgen.1002280 (PMC3174201; doi:10.1371/journal.pgen.1002280)
Supplement: Table S11 — Laboratory assessment of the father. (DOC) [file pgen.1002280.s016.doc]

**Table S11**. Laboratory assessment of the father

| Test | Value | Reference range | Units |
| --- | --- | --- | --- |
| CBC | | | |
| White blood cells | 4.1 | 4.5-11.0 | K/uL |
| Hemoglobin | 13.5 | 13.9-16.3 | g/dL |
| Hematocrit | 39.8 | 39.0-55.0 | % |
| Mean corpuscular volume | 89.0 | 80.0-100.0 | fL |
| Platelet count | 203 | 150-450 | K/uL |
| Coagulation | | | |
| Prothrombin time* | 27.5 | 9.7-12.8 | Seconds |
| INR* | 2.4 | 2.0-3.0 | -- |
| Activated partial thromboplastin time | 35.0 | 25.0-36.8 | Seconds |
| Fibrinogen | 272 | 180-445 | mg/dL |
| Blood type | A positive | -- | -- |
| Serum chemistry and immunology, fasting | | | |
| Sodium | 141 | 135-145 | mmol/L |
| Potassium | 4.3 | 3.5-5.0 | mmol/L |
| Chloride | 103 | 101-111 | mmol/L |
| Bicarbonate | 31 | 22-32 | mmol/L |
| Glucose | 97 | 70-100 | mg/dL |
| BUN | 18 | 8-20 | mg/dL |
| Creatinine | 0.9 | 0.7-1.2 | mg/dL |
| Calcium | 9.2 | 8.5-10.5 | mg/dL |
| Total protein | 6.6 | 6.0-8.5 | g/dL |
| Albumin | 3.8 | 3.0-5.0 | g/dL |
| Bilirubin, total | 0.6 | 0.2-1.3 | mg/dL |
| Alkaline phosphatase | 64 | 38-126 | IU/L |
| AST | 30 | 15-41 | IU/L |
| ALT | 29 | 11-63 | IU/L |
| Homocysteine | 11.4 | 0-12.5 | umol/L |
| Total cholesterol | 178 | 0-199 | mg/dL |
| Triglycerides | 119 | 20-150 | mg/dL |
| LDL | 102 | <130 | mg/dL |
| HDL | 52 | >35 | mg/dL |
| Lipoprotein (a) | 1 | 0-30 | mg/dL |
| Hemoglobin A1C | 5.3 | 4.6-6.2 | % |
| Prostate specific antigen | 1.26 | 0.0-4.0 | ng/mL |
| Urine chemistry | | | |
| Cysteine | 40 |  |  |
| *Obtained while on warfarin therapy | | | |
