# Supplementary material for: Phased Whole-Genome Genetic Risk in a Family Quartet Using a Major Allele Reference Sequence
Source: PLoS Genet. 2011 Sep 15;7(9):e1002280. doi: 10.1371/journal.pgen.1002280 (PMC3174201; doi:10.1371/journal.pgen.1002280)
Supplement: Text S1 — Supplementary materials and methods. (DOC) [file pgen.1002280.s017.doc]

**Text S1 – Supplementary Materials and Methods**

I. Synthetic major allele reference genomes [2](#__RefHeading___Toc173237067)

Data sources [2](#__RefHeading___Toc173237068)

Synthetic major allele reference sequence generation [2](#__RefHeading___Toc173237069)

II. Subject and sample characteristics [3](#__RefHeading___Toc173237070)

Study subjects [3](#__RefHeading___Toc173237071)

III. Genome sequencing, mapping, and base calling. [4](#__RefHeading___Toc173237072)

Genomic DNA isolation and sequencing library preparation [4](#__RefHeading___Toc173237073)

Sequence generation [4](#__RefHeading___Toc173237074)

Sequence alignment and mapping [4](#__RefHeading___Toc173237075)

Base calling and variant calling [5](#__RefHeading___Toc173237076)

Genetic variant quality control [6](#__RefHeading___Toc173237077)

Error rate estimation [7](#__RefHeading___Toc173237078)

IV. Inheritance state analysis and phasing [8](#__RefHeading___Toc173237079)

Family inheritance state analysis [8](#__RefHeading___Toc173237080)

Phasing [10](#__RefHeading___Toc173237081)

Immunogenotyping [12](#__RefHeading___Toc173237082)

V. Ancestry analysis [13](#__RefHeading___Toc173237083)

Principle components analysis of ancestry [13](#__RefHeading___Toc173237084)

VI. Rare and novel genetic variant risk prediction [13](#__RefHeading___Toc173237085)

Definitions and heuristic [13](#__RefHeading___Toc173237086)

Nonsynonymous coding variants [14](#__RefHeading___Toc173237087)

Synonymous coding variant risk prediction [17](#__RefHeading___Toc173237088)

Noncoding variant risk prediction [22](#__RefHeading___Toc173237089)

Structural variant risk prediction [22](#__RefHeading___Toc173237090)

VII. Common genetic variant risk prediction [23](#__RefHeading___Toc173237091)

Quantitative disease-SNP association database [23](#__RefHeading___Toc173237092)

Calculation of predicted personal genetic risk for 28 common diseases [23](#__RefHeading___Toc173237093)

Calculation of relative population based disease risk [24](#__RefHeading___Toc173237094)

Family differential risk and parental contribution to common disease risk [24](#__RefHeading___Toc173237095)

VIII. Pharmacogenomics [25](#__RefHeading___Toc173237096)

Pharmacogenomics Knowledge Base [25](#__RefHeading___Toc173237097)

Variant level annotation [25](#__RefHeading___Toc173237098)

IX. Clinical assessment [25](#__RefHeading___Toc173237099)

Laboratory testing [25](#__RefHeading___Toc173237100)

XII. References [26](#__RefHeading___Toc173237101)

#### I. Synthetic major allele reference genomes

##### Data sources

The 1000 genomes project has provided an extensive catalog of genetic variation in human populations, allowing for fine-scale mapping of variation in several different ethnic groups. Data regarding allele frequencies in each of the three major Haplotype Map (HapMap) populations was obtained from genotypes in the low coverage whole genome sequencing pilot (pilot 1) of the 1000 genomes project (12/13/2010 release). This compendium catalogs variation at 7,917,426, 10,903,690, and 6,253,467 sites in the CEU, YRI, and CHB/JPT populations, respectively, with sensitivity for an alternative allele of > 99% at allele frequencies > 10%.

##### Synthetic major allele reference sequence generation

We first identified genomic positions corresponding to NCBI reference genome sequence 37.1 coordinates at which the major allele (defined as estimated allele frequency > 50%) differed from the reference sequence base. We then substituted the major allele for each of the three HapMap populations for the reference base to create three synthetic ethnicity-specific major allele references, specific to each of the CEU, YRI, and CHB/JPT populations. This resulted in a reference base change at 1,543,755, 1,658,360, and 1,676,213 positions in the CEU, YRI, and CHB/JPT populations, respectively. As demonstrated in **Figure 2**, there were 796,548 positions common to all three population groups at which the major allele differed from the NCBI reference base. The YRI population had the largest proportion of base changes with a major allele not corresponding to either of the two other population groups. The frequency with which the reference base differed from the ethnicity-specific dominant base was relatively consistent across autosomes, with the exception of chromosome 6, which demonstrated increased sequence divergence in the reference genome from each of the three dominant population alleles in the vicinity of the HLA locus. The major allele reference sequences are freely available upon request from the authors.

#### II. Subject and sample characteristics

##### Study subjects

Clinical characteristics of study subjects are described in graphical form in the pedigree in **Figure 1**. One study subject (the father) had a history of recurrent venous thromboembolism and hyperlipidemia and was taking warfarin for anticoagulation, the lipid lowering medications ezetemibe and simvastatin, and the proton pump inhibitor esomeprazole. Two family members had a history of psoriasis (father and daughter). Two family members had a history of allergic rhinitis (mother and son) and were taking loratidine on an as needed basis. Both parents self-reported northern European ancestry.

The study was approved by the Stanford University Institutional Review Board and all study subjects attended genetic counseling and provided informed written consent (or assent, in the case of the children). This consent process occurred at two points in time: before the sequencing was performed (overseen by Illumina, Inc., and conducted with a clinical geneticist) and before this clinical interpretation was performed (conducted with a genetic counselor and research assistant). Pedigree and genotyping results were discussed in a genetic counseling session in the context of information that may be obtained in a clinical interpretation of genome sequence data and the personal and family risks and benefits that may arise in obtaining this information.

#### III. Genome sequencing, mapping, and base calling.

##### Genomic DNA isolation and sequencing library preparation

Peripheral blood was obtained from study subjects and sample DNA was directly isolated from according to standard protocols. Genomic DNA was fragmented by sonication and sequencing libraries were prepared by clonal amplification, end repair, and sequencing adapter ligation according to standard Illumina protocol by Illumina, Inc. (San Diego, CA).

##### Sequence generation

Illumina Inc. performed all sequencing reactions on the GA II instrument using massively parallel reversible terminator (sequencing by synthesis) chemistry to generate 75 base pair paired-end reads. Sequence reads passing standard Illumina GA pipeline filters were retained for further analysis.

##### Sequence alignment and mapping

As pilot alignments to chromosomes 6 and 22 in one individual demonstrated no large difference in alignment efficiency between HG19 and the CEU major allele reference (**Table S2**), paired-end short reads were aligned to NCBI reference genome build 37.1 (obtained from the UCSC Golden Path genome browser) using the Burrows-Wheeler Aligner (BWA) software version 0.5.8a. Reads were trimmed to a length of 35 base pairs if the phred-like quality score was less than 20; default settings were otherwise used, resulting in sequence alignment map format (SAM) alignment files for paired end reads. We next used software from the Genome Analysis Tool Kit (GATK) version 1.0.4075 to remove PCR duplicates and perform sample-level local realignment around known indels. Finally, mate pair information was re-synced using the Picard FixMateInformation tool and base quality score recalibration was performed using GATK, producing an aligned, cleaned binary alignment format (BAM) file for each study subject.

##### Base calling and variant calling

We used the samtools multi-sample pileup tool with default settings to compute likelihoods of observed base data at each covered position given each possible underlying genotype. BCFtools was used to apply prior probabilities of each genotype and perform base calling against both the NCBI reference genome version 37.1 and the CEU major allele reference genome sequence created as described above. Single nucleotide variants were identified at an average distance of 699 base pairs when compared with the NCBI reference and 809 base pairs when compared with the CEU major allele reference. Short indels were called concurrently with single nucleotide variants using the samtools multi-sample pileup tool. As described in **Figure S1**, this resulted in genotype calls at 91.7%, 92.3%, 92.4%, and 92.4% of all chromosomal positions in the mother, father, son, and daughter, respectively. Across the quartet 91.6% of chromosomal positions were genotyped in all four family members. Haploid depth of coverage was 37.3x in the mother, 37.1x in the father, 46.2x in the son, and 36.2x in the daughter (**Figure S1**). At 3,858 variant positions the genotype for at least one family member differed when compared to the HG19 and CEU major allele references, most frequently in the vicinity of indels. Two loci at rs757210 and rs1553318 had known disease associations in genome wide association studies meeting the criteria for inclusion in common variant risk analysis outlined below. In both instances the genotypes in the variant calls against the CEU major allele reference were concordant with previously observed alleles reported in dbSNP 132, while the variant alleles in calls against the HG19 reference sequence were not reported previously. Across the family quartet the calculated transition to transversion ratio (Ti/Tv) in the final call set was 2.49, which corresponds to previous estimates of expected Ti/Tv.

##### Genetic variant quality control

We used both orthogonal genotyping technology and bioinformatics tools to perform variant quality control and exclude likely spurious genotype calls. For confirmatory testing of common variants and quality score calibration, we genotyped genomic DNA isolated from saliva from all four study subjects using a customized array built on the Illumina HumanHap 550K+ Genotyping BeadChip (23andME, Inc., Mountain View, CA). This genotyping array contains probesets corresponding to approximately 578,000 single nucleotide variants, including ~30,000 variants unique to the 23andME array implementation. We calculated the discordance rate between array-based genotyping and sequencing variants according to mapping quality, base depth, and genotype quality. As described above, reads not meeting a threshold for the base quality score were trimmed at the stage of initial BWA mapping. All discordant calls were excluded from risk interpretation of genetic variants, as neither technology (array based genotyping or whole genome sequencing) was considered the gold standard. This information was also subsequently used to choose quality score cutoffs as follows: variants were retained if the mean mapping quality was greater than 40, the average base coverage depth was greater than 10, and the average and minimum genotype qualities were greater than 45 and 15, respectively.

We also leveraged the information provided by family-based sequencing for quality control of variant calls. In a family quartet, of the 81 possible genotype combinations in a bialleleic state, 52 correspond to Mendelian inheritance errors (MIEs), or allele assortments that are impossible under Mendel’s laws. A very small subset of these allele assortments will be due to germ-line or somatic de novo mutation events, gene conversions, or hemizygous structural variation. However, the overwhelming majority of these allele assortments result from sequencing errors. Therefore, the identification and sequestering of these variants can greatly reduce the genotyping error rate. Similarly, the identification of regions of the genome that are prone to sequencing errors or errors in mapping and consensus assembly due to discordant structural variation between the reference and sample sequence can greatly reduce the error rate. Lastly, the identification of allele assortments discordant with the neighboring inheritance state (state consistency errors, SCEs) allows for identification of sequencing errors. We thus excluded all MIEs and SCEs from subsequent annotation, as well as all variants in error prone regions.

##### Error rate estimation

We estimated genotyping error rate via three methods: 1) estimation of the MIE rate per base sequenced, 2) estimation of the MIE and SCE rate in the 24% of the genome in which the children were identical by descent, and 3) estimation of the discordant rate between the 23andME genotyping and the whole genome sequence call. All three methods yielded approximately the same error rate at each stage of variant quality control. As demonstrated in **Figure 3,** the greatest reduction in error rate occurred with filtering of variants in error prone regions, with a final estimated error rate by MIE rate per base pair sequenced of 2.1 x 10^-6^ for the CEU and HG19 variant call sets. The overall SCE rate per base pair sequenced in identical-by-descent regions for the offspring was 5.26 x 10^-7^ for both CEU and HG19 call sets. This represents a 94% overall reduction in error rate using a combination of orthogonal genotype confirmation and filtering of error prone regions.

#### IV. Inheritance state analysis and phasing

##### Family inheritance state analysis

We applied the concept of inheritance state developed by Roach, et al, to the allele assortments resulting from single nucleotide variants in each of the four family members. This nuclear family of four has 4 possible inheritance states: maternal identical, in which the children each inherit the same allele from the mother, paternal identical, in which the children each inherit the same allele from the father, identical, in which the children are identical by descent and inherit the same allele from both parents, and nonidentical, in which the children inherit different alleles from both parents. We used two algorithms to determine inheritance state for neighboring SNVs. The first heuristic algorithm binned allele assortments into 100 kb pair regions based on chromosomal position and assigned an inheritance state according to the total number of SNPs in the bin consistent with that inheritance state.

The second algorithm was based on a Hidden Markov Model (HMM) in which the hidden states correspond to the four inheritance states described above and two error states first described by Roach, et al. These two states were the compression/CNV state, in which hemizygous structural variants in the study genomes or reference genome result in uniform heterozygosity across the quartet, and the MIE-rich state, which contains a high number of impossible allele assortments likely due to sequencing or assembly errors. The emission probabilities for allele assortments consistent with each inheritance state were set equal to one another and the total probability of emitting an inconsistent allele assortment was set to 0.005. For the CNV/compression state, the emission probability for uniform heterozygosity was set to 0.66. The MIE-rich state was modeled to emit an MIE 33% of the time and a consistent allele assortment 67% of the time, with equal probability weight for each consistent allele assortment. Transition probabilities for each of the four non-error and two error inheritance states were set according to the expected number of state transitions and the total number of allele assortments in the quartet, with the remaining transition probability allocated to self-self transitions. Manipulation of these transition probabilities within four orders of magnitude did not qualitatively change the resulting inheritance state determination. The Viterbi algorithm was used to find the most likely state path given the observed allele assortments, resulting in the assignment of an inheritance state to each allele assortment in which one or more family members had an allele differing from the reference sequence. State transitions in this path correspond to recombination events, with recombination window resolution given by the distance between informative allele assortments.

As CNV/compression regions and MIE-rich regions are potential sources of spurious state switches and, therefore, incorrectly inferred recombination events, the identification of these regions can improve recombination inference accuracy. After excluding SNVs in these regions, a four state HMM was developed and the viterbi path again found, resulting in an improved median recombination window resolution 963 base pairs. To identify enrichment for these recombination windows within known hotspots, a quantitative trait associated with *PRDM9* allele status, we calculated the number of recombinations in which a maximum recombination rate of > 10 cM/Mbp was observed. We employed a Monte Carlo simulation with 10,000 replicates to estimate the hotspot enrichment for 106 randomly placed recombination windows of width equal to that observed in the quartet, finding that 4.1% of these random windows were in hotspots. Given that we found 52 recombination windows in hotspots in the quartet, this corresponds to a *p* value for hotspot enrichment of 2.0x10^-73^.

##### Phasing

Haplotype phase is important to understanding genetic risk in patients with and without disease phenotypes. Resolution of genotype information provided by whole genome sequencing into phased haplotypes has long proved difficult. A variety of statistical phasing techniques exist for inferring haplotype phase in unrelated individuals that seek to minimize recombination events and/or maximize the likelihood of heterozygous positions in high linkage disequilibrium assorting together on contigs. These techniques do not provide long-range phasing, however, precluding assessment of multigenic contribution to disease phenotypes or assessment of parental contribution to risk profiles. Father-mother-child trio sequencing provides information on long-range haplotype phase but only provides definitive phasing information at ~80% of heterozygous positions, as uniformly heterozygous positions are not informative. Sequencing an additional sibling allows for precise identification of meiotic crossovers, as well as a framework for understanding the inheritance state of contiguous polymorphic markers. We used a combination of pedigree data and statistical phasing based on inheritance state and, for uniformly heterozygous positions, population linkage disequilibrium data to determine long-range haplotypes for the family quartet (heuristic described in **Figure 3**). The phase of approximately 84% of heterozygous positions in each child could be resolved by pedigree data alone. The inheritance state of the surrounding variants was used to phase 11% of the remaining heterozygous positions. This information was most informative for positions at which each of three individuals in a father-mother-child trio was heterozygous for a non-reference allele and the sibling was homozygous for the reference or non-reference allele. For uniformly heterozygous positions in which the family information is not informative, we used pair-wise pre-computed population linkage disequilibrium data from the SNP Annotation and Proxy Search (SNAP) database to assign the minor allele to the paternal or maternal chromosome scaffolds according to maximization of aggregate r^2^. Specifically, we considered SNPs genotyped in the HapMap II and III CEU populations with r^2^ values > 0.3 within 250 kb of the uniformly heterozygous position for this analysis given the inconsistent quality of linkage data outside this window. For each haplotype scaffold $h_{m}$and $h_{p}$ and uniformly heterozygous locus *l,* we calculated the likelihood of *l* residing on *h* as:

(1)

where *n* is the number of heterozygous loci on *h*, and $r_{i}^{2}$ is the value for r^2^ between the minor allele at *l* and the minor allele at heterozygous loci *i* on *h*. We then placed the minor allele at *l* on haplotype scaffold$h_{m}$or$h_{p}$ according to.

This methodology allowed us to phase 34% of the remaining uniformly heterozygous positions. Finally, phasing was performed for each adult in contigs according to passage of allele contigs to one, both, or neither of the children. Phasing of adult contigs across recombination sites was not attempted due to largely uninformative linkage disequilibrium structure across recombination sites, which fell into areas of high population-averaged recombination rates. This combination of pedigree and population-linkage disequilibrium-based phasing resulted in phase resolution of 97.9% of heterozygous positions; family information alone informed phasing of 96.8% of positions.

##### Immunogenotyping

Phased genetic variant information simplifies greatly the combinatorial problem of resolving clinically relevant haplotypes in genomic locations with high recombination rates, in which traditional population-based statistical methods for haplotype determination are most problematic. The Human Leukocyte Antigen Loci (HLA) are examples of such regions. We used a combination of phased haplotype information for each family member and known tag haplotypes to estimate HLA type based on genotype for all four family members. We performed a leave-one-out iterative search using the phased hapotype information from each family member for the nearest common tag haplotype for HLA type, assigning the HLA type for each chromosome (paternal or maternal origin for the children, and contigs transmitted to one, both, or neither of the children in adults). This resulted in HLA types for each haploid chromosome 6 as displayed in **Figure 3**.

#### V. Ancestry analysis

##### Principle components analysis of ancestry

To determine the ancestral origins for the quartet, we performed principal components analysis (PCA) using the maternal and paternal genotypes and a subset of individuals of European ancestry from the Population Reference Sample (POPRES) data set. Briefly, we combined maternal and paternal genotypes with the genotypes from 10 individuals from Eastern/Southeastern Europe, 190 individuals from central Europe, 78 individuals from Northern/Northeastern Europe, 167 individuals from Northwestern Europe, 238 individuals from Southern Europe, 99 individuals from Southeastern Europe, 272 individuals from Southwestern Europe and 403 individuals from Western Europe (EuropeESE, EuropeC, EuropeNNE, EuropeNW, EuropeS, EuropeSE, EuropeSW, and EuropeW respectively in **Figure 4**). Next, we used PLINK to filter SNPs with greater than 10% missing data and a minor allele frequency less than 10%, and thinned 50-SNP windows to remove pairs of SNPs with an R^2^ greater than 0.9. With the remaining 124,378 SNPs we used EIGENSTRAT to perform a principal components analysis. Principle components one and two recapitulate the structure of continental Europe and reveal maternal and paternal ancestry to be North/Northeastern and Western European respectively.

#### VI. Rare and novel genetic variant risk prediction

##### Definitions and heuristic

The operational definition of a rare variant was a variant with an allele frequency of less than 5%. We used a hierarchical search for allele frequencies that first considered ethnicity-specific allele frequencies and then population-wide allele frequencies as follows:

1. Ethnicity-specific allele frequencies from the dbSNP 132 database
2. Ethnicity specific allele frequencies from the 1000 genomes pilot 1 data
3. Allele frequencies from the dbSNP 132 full database
4. Allele frequencies from the 1000 genomes full project data 12/13/2010 release

Alleles with an assigned rsid but no published allele frequency were considered rare but with no frequency data, and alleles not found in any published database were considered novel. This resulted in sets of 351,555 and 354,074 rare or novel variants from the HG19 and CEU reference variant call sets, respectively.

The overall heuristic for searching novel and rare variants for significant disease associations is presented in **Figure S3.** We used the CCDS collection of coding sequence positions to assign rare and novel variants to coding and noncoding categories. We next annotated putative rare and novel loss of function variants in coding and noncoding regions of genes known to be associated with Mendelian diseases as defined by the Online Mendelian Inheritance in Man database.

##### Nonsynonymous coding variants

Non-synonymous variants (nsSNPs) were annotated using a combination of prediction algorithms and manual curation. All rare and novel variants were annotated using the Sorting Intolerant from Tolerate (SIFT) algorithm developed by Henkoff, et al, which predicts the effects of non-synonymous polymorphisms on protein function based on homology, conservation, and physical properties of amino acid substitutions. We used the Polymorphism Phenotyping (PolyPhen) 2 tool in parallel to annotate coding regions. This algorithm is a probabilistic classifier that predicts the impact of amino acid changes on protein function using an algorithm that incorporates information on site of substitution (whether an amino acid change occurs in one of several sites of functional importance such as binding sites or trans-membrane regions), multiple sequence alignment, and known protein structural changes. For this analysis we used the human variation trained data, which is based on known damaging alleles involved in Mendelian diseases and multiple sequence alignment with closely related mammalian homologs. This training set has superior performance for distinguishing variants with severe effects from more abundant, mildly deleterious variants.

Because the prediction accuracies of SIFT and PolyPhen have been shown to depend heavily on the evolutionary anatomy of genomic position at which an nsSNP occurs, we further annotated nsSNPs using position-specific evolutionary features derived from a 46 species multiple sequence alignment (MSA) of vertebrate genomes obtained from the UCSC Genome Browser (http://genome.ucsc.edu/; Accessed November 30, 2010). For each genomic position, we computed the evolutionary rate (mutations per billion years) across the mammalian lineage using the method previously described by Fitch, and also computed the evolutionary time-span (ETS) as the proportion of non-gap sequences in the MSA at a position. For coding variants of unknown significance, the mammalian evolutionary rate is proportional to the fraction of selectively neutral alleles at a position, and can therefore serve as a prior expectation in determining the likelihood that an observed nsSNP is deleterious. In general, SNVs found at positions having a mammalian evolutionary rates > 1 are not likely to be associated with deleterious protein sequence changes, and SNVs with evolutionary rates > 2 are highly unlikely to be deleterious, as most “neutral” population variation characterized by HapMap and other population samples lies in this range. Consequently, the prediction accuracies of SIFT and PolyPhen are substantially reduced at positions having an evolutionary rate > 1.

In order to prioritize the evaluation and delivery of information relating to the rare and novel variants, we developed and applied a rating schema based on phenotype-level information about the variants in Mendelian-disease associated genes and predicted or experimentally derived variant pathogenicity. This rating schema is summarized in **Table S4**. Of 200 novel or rare non-synonymous variants coding variants associated with OMIM-disease genes, 72 were in genes known to be associated with plausible Mendelian diseases. These variants were manually curated according to this rating schema using a combination of private and public mutation database data including the Human Gene Mutation Database, dbSNP, Entrez Gene, and disease specific databases with follow up review of the primary literature when available. Of these 72 manually curated variants, five were associated with known disease phenotypes. The remaining 67 variants were of unknown significance, and were rated according to predicted pathogenicity, disease phenotype, and position-specific rates and the evolutionary time span and are displayed in **Table S5**.

The children were compound heterozygous for variants in four disease-related genes: *BLM* (mother, son and daughter)*,* associated with Bloom syndrome, *MLH3* (son)*,* associated with familial non-polyposis colon cancer, *SLC4A5* (son)*,* associated with proximal renal tubular acidosis, and *COG7* (daughter)*,* associated with type IIe congenital disorder of glycosylation. There were two instances of homozygosity for rare/novel variants in disease-related genes in the daughter (*KRT8,* associated with monilethrix, and *ASAH1*, associated with Farber lipogranulomatosis) and one such instance in the son (*PLEC1,* associated with epidermolysis bullosa simplex, **Table S6**). With the exception of hereditary non-polyposis colon cancer (HNPCC), all of these conditions present in infancy or early childhood and therefore the lack of associated trait phenotypes brings into question the associated variant-level pathogenicity. One of the *MLH3* variants is predicted to be benign by both prediction algorithms and has a high evolutionary rate, providing further evidence that it is likely a benign, albeit rare, polymorphism. The other variant in *MLH3* has no known clinical association, is described as a “natural variant” the UniProt database, and is predicted to be tolerated by SIFT but possibly damaging by PolyPhen2, and has a low evolutionary rate. It is unclear what role these variants may play in predisposition to HNPCC, but the lack of any of the major criteria for diagnosis of this autosomal dominant condition suggests that they are not likely to be causative for HNPCC.

##### Synonymous coding variant risk prediction

Apart from amino-acid substitutions, there are several ways that synonymous single nucleotide polymorphisms (sSNPs) can affect a gene and its resulting protein products. Alteration of splice sites can modify how a gene is spliced and result in important changes in the resulting mRNAs; most of these alterations result in premature mRNA degradation. Creation of spurious splice sites may affect the resulting protein sequence. Other factors that affect protein production and structure include mRNA decay rates and mRNA structural motifs surrounding important regulatory sites (such as 5' and 3' UTRs). Finally, codon usage bias can have a direct effect on protein elongation and translational kinetics, a consequence of the correlation between codon usage frequency and tRNA availability. This gives us three main mechanisms that we can computationally explore to detect putative phenotypic changes provoked by sSNPs (**Figure S4**).

Aberrant splicing is a phenomenon that has been linked to synonymous mutations in various studies. Creation and disruption of 5' donor splice sites and exonic splice site enhancers through synonymous alterations have been reported to be part of the etiology of diseases such as type 1 neurofibromatosis, multiple sclerosis, and phenylketonuria. Many splice site prediction algorithms already exist and are primarily used for genome-wide gene detection of splice sites. However, they can also be used to detect putative disruption or creation of splicing sites in a simplistic fashion: by comparing predictions when applying the algorithm to reference and the variant DNA sequences. Using this criteria, we applied the maximum entropy splice site detection algorithm to the flanking sequence of a sSNP with and without the polymorphic substitution. Predictions resulting in a positive odds ratio for the reference sequence but in a negative odds ratio for the sequence with the polymorphism are flagged as putative splice site disruptions. Conversely, a combination of a negative prediction for the reference sequence and a positive score for the SNP-affected sequence is reported as putative creation of a splice site.

Several mRNA structural factors are associated with important effects on phenotype. Secondary structure may directly affect mRNA decay rates as well as confer protection from premature degradation. Furthermore, highly structured UTRs can prevent regulatory molecules, such as microRNAs, from performing proper regulatory functions. Thus, investigating the effects of sSNPs in mRNA structure becomes a great pivotal point to indirectly study putative changes in the resulting protein. A small set of articles have already laid ground on the case, by analyzing the influence of sSNPs in mRNA secondary structure.

RNA secondary structure prediction is a classical problem in computational biology and there are many methods that give reasonable estimates. Most of them report the resulting free energy, , of the predicted secondary structure, thereby giving a thermodynamic measure of structure. Algorithms for detecting non-coding RNAs use free energy along with other heuristics to detect putative biologically active transcripts. In particular, these algorithms attempt to find a 'structural signal' in a certain window of nucleotides while scanning a genome. One approach to do this is by performing free energy calculations for randomized samples of the same size and monomeric or dimeric conformations than that of the current window. A Z-score is then given to the window, defined as:

(2)

where $G(\text{seq})$ is the free energy of the RNA sequence , $G_{m}(\text{seq},S)$ is the average free energy of the sequences of the sample set$S$ that have the same length and monomeric (or dimeric, if desired) conformation than , and $G_{s}(\text{seq},S)$ is the standard deviation of the free energies of . There has been evidence demonstrating that secondary structure by itself does not give a strong signal from random sequences with the same monomer or even dimer conformations. We argue that this is indeed expected, since permutation of nucleotides is a far more benign alteration than deletion, insertion, or replacement. To express this in the Z-score, we modify the definition of the sample set to a set of random sequences of the same length of the window but not necessarily with the same n-meric conformation.

To apply the Z-score notion to probe if a change in secondary structure occurs with a SNP, we decided to assess the structural significance of the subsequence flanking the SNP. We did this by taking two windows: the flanking window $W_{f}$ and the sampling window $W_{s}$. The flanking window is the sequence that contains the SNP position in its midpoint. The sampling window is a subsequence of the flanking window and also contains the SNP position. We then perform the sampling from the set $S(W_{f},W_{s})$ of sequences with length of the flanking window that vary only in the sampling window. Finally we take the Z-score, as defined previously in equation 3, using this sample set:

(3)

This is done using the ViennaRNA folding package. We then compare the Z-score of the reference sequence with the Z-score of the sequence containing the SNP substitution and obtain a difference score. This score expresses the difference between structural importance of the sequence in the sampling window in the reference and SNP-containing sequence.

Two genes that code for the same protein using synonymous codons do not necessarily give the same result. This is mainly due to the fact that tRNA iso-acceptors do not have equal abundance in the cell. Even though this statement was confirmed *in vitro* several years ago, only recently have we seen such a situation occurring *in vivo*. The demonstration that codon usage bias can alter translational kinetics opens an interesting new venue to search for relations between phenotype alterations and sSNPs. Codon usage bias analysis is not new, and has been fairly well studied since the beginning of the genomic era. Several results confirm that, in some organisms, codon usage is also related with position, since it is not rare to see codons with similar relative frequency (relative frequency is the frequency of a codon occurring in a genome with respect to codons that code for the same amino-acid while absolute frequency is the frequency of codon occurrence with respect to the set of all codons) cluster together in particular sites. This has led to the speculation that codon choice is directed by evolution, given that there could be selection constraints acting in some aspects of translational kinetics, such as protein elongation. Following this conceptualization, we assess changes in codon bias via a clustering criterion. Given an exon sequence , we first produce a set of pairs for all possible *n* in *seq*, where *n* is the *n*^th^ codon in the sequence given the *i*^th^ open reading frame, *N* is the total number of codons in the sequence, and $\text{rel}_{n}$ is the relative frequency of the *n*^th^ codon. We then apply the *k*-means clustering algorithm to $C_{i}(\text{seq})$ for each open reading frame (ORF) with a given *k*. This is performed with both the reference and SNP-modified sequence, . Finally, we compare, for all ORFs, the resulting centroids between both sequences and compute the sum of their distances, taking the minimum of these values. In other words, the final codon usage score is:

(4)

where $C_{k,i}$ is the set of *k* centroids in the *i*^th^ ORF.

##### Noncoding variant risk prediction

We searched for rare and novel variants in noncoding regions associated with introns, 3’ and 5’ UTRs, and miRNA target regions in 3’ UTRs of genes associated with Mendelian disorders as well as pre-miRNA and mature miRNA sequences targeting genes associated with Mendelian disorders. miRNA target regions and sequence coordinates were obtained from the miRbase database. As a supplement to the maximum entropy splice site disruption algorithm described above, we also searched known splice donor and acceptor sites for rare and novel variants. Rare and novel variants in pre-miRNA, mature miRNA, miRNA target regions, and splice sites were annotated as putative loss of function variants.

##### Structural variant risk prediction

Indels were annotated based on their rarity (with allele frequencies derived from the 1000 genomes pilot 1 data), association with coding regions, whether they disrupted a splice site, and whether they were predicted to cause a frame-shift in an open reading frame. As allele frequencies for indels are less reliable than for single nucleotide variants, we considered novel frameshift indels in coding regions or novel indels in splice sites of genes associated with OMIM-curated diseases to be loss of function variants. There were 27 such variants when compared to the HG19 reference genome and 29 such variants when compared to the CEU major allele reference genome.

#### VII. Common genetic variant risk prediction

##### Quantitative disease-SNP association database

As described previously, we manually curated quantitative human disease-SNP associations from the full text, figures, tables, and supplemental materials of 4,022 human genetics papers, and recorded more than 100 features from each paper, including the disease name (e.g. coronary artery disease), specific phenotype (e.g. acute coronary syndrome in coronary artery disease), study population (e.g. Finnish individuals), case and control population (e.g. 2,508 subjects with coronary artery disease proven by angiography), gender distribution, genotyping technology, major/minor risk alleles, odds ratio, 95% confidence interval of the odds ratio, published p-value, and genetic model. Studies on similar diseases were categorized and mapped to the Concept Unique Identifiers (CUI) in the Unified Medical Language System (UMLS). For each study, the frequency of each genotype and allele in the case and control populations was recorded. Strand ambiguities were resolved with an automatic strand detection algorithm described previously.

##### Calculation of predicted personal genetic risk for 28 common diseases

For each of 28 diseases we identified all SNPs that had been significantly associated with the disease with a p-value of ≤10^-6^ in two or more Genome-Wide Association Studies (GWAS) with a total sample size of 2,000 or more subjects. We estimated genetic risk using a likelihood ratio for each SNP defined by the relative frequency of the individual’s genotype in the diseased vs. healthy control populations (e.g., given an allele “A”, LR = Pr(A|diseased)/Pr(A|control)). The LR incorporates both the sensitivity and specificity of the test and provides a direct estimate of how much a test result will change the odds of having a disease. We excluded studies with diseased subjects in the control group and studies on non-Caucasian populations. For each SNP, we averaged the LRs from multiple studies with a weight of the square root of the sample size to give higher confidence to studies with larger sample size. After removing SNPs in linkage disequilibrium (R^2^≥0.3 in the corresponding population group), we assumed each locus as an independent genetic test and multiplied LRs to report the summarized score or predicted genetic risk. Pre- and post-test estimates of disease risk were calculated for the father using age and sex-matched cohorts for estimation of pre-genotype disease risk and the composite likelihood ratios for post-genotype disease risk.

##### Calculation of relative population based disease risk

To evaluate the relative population based disease risk, we calculated the personal genetic risk on 58 diseases for 174 CEU individuals from the HapMap project version II and III (**Figure 5**). Only SNPs that were genotyped in both CEU and the family quartet were included. The disease risk percentile score was calculated as the percentage of CEU individuals who have a lower risk than the subject in question.

##### Family differential risk and parental contribution to common disease risk

We calculated the disease risk for each of the family members according to phased SNP genotypes as described above; this information is displayed in **Figure 5.** For the daughter and son, we calculated the paternal and maternal disease risk allele contribution according to likelihood ratios from phased variant genotypes at each SNP locus, generating an estimate of paternal and maternal haplotype disease risk contribution in each child (**Figure 5**).

#### VIII. Pharmacogenomics

##### Pharmacogenomics Knowledge Base

We annotated genome-wide pharmacogenomic associations using the Pharmacogenomics Knowledge Base (www.pharmgkb.org), an online pharmacogenomics resource containing manually created annotations on a large collection of pharmacogenomics literature. We curated over 1400 drug-variant-phenotype relationships from the literature and used these relationships to create clinical annotations for 298 known variants. Some variants have more than one clinical annotation to represent different drug-phenotype relationships.

##### Variant level annotation

Drug-variant phenotypes can be very complicated and specific to certain patient populations. For simplicity and illustration purposes, clinical annotations were binned into the following groups: Drug(s) More Likely to Work, Drug(s) Less Likely to Work, Drug(s) More Likely to Cause Side Effect, Drug(s) Less Likely to Cause Side Effect, Drug Dose(s) Easy to Predict, Drug Dose(s) Difficult to Predict, Drug Dose(s) Above Average, Drug Dose(s) Below Average, No Pharmacogenomic Action and/or Phenotype Unknown and/or Phenotype Not Applicable (example: ovarian cancer risk for males). An example annotation is given in **Table S7** and full annotations for the study subjects are provided in **Tables S8, S9,** and **S10**.

#### IX. Clinical assessment

##### Laboratory testing

We performed laboratory assessment of the father guided by genetic risk assessment. Results of laboratory testing are presented in **Table S11**.

#### XII. References

1. Durbin RM, Abecasis GR, Altshuler DL, Auton A, Brooks LD, et al. (2010) A map of human genome variation from population-scale sequencing. Nature 467: 1061-1073.

2. Ormond KE, Wheeler MT, Hudgins L, Klein TE, Butte AJ, et al. (2010) Challenges in the clinical application of whole-genome sequencing. Lancet 375: 1749-1751.

3. Bentley DR, Balasubramanian S, Swerdlow HP, Smith GP, Milton J, et al. (2008) Accurate whole human genome sequencing using reversible terminator chemistry. Nature 456: 53-59.

4. Li H, Durbin R (2009) Fast and accurate short read alignment with Burrows-Wheeler transform. Bioinformatics 25: 1754-1760.

5. Nachman MW, Crowell SL (2000) Estimate of the mutation rate per nucleotide in humans. Genetics 156: 297-304.

6. Roach JC, Glusman G, Smit AF, Huff CD, Hubley R, et al. (2010) Analysis of genetic inheritance in a family quartet by whole-genome sequencing. Science 328: 636-639.

7. Williams AL, Housman DE, Rinard MC, Gifford DK (2010) Rapid haplotype inference for nuclear families. Genome Biol 11: R108.

8. Marchini J, Howie B, Myers S, McVean G, Donnelly P (2007) A new multipoint method for genome-wide association studies by imputation of genotypes. Nat Genet 39: 906-913.

9. Kruglyak L, Daly MJ, Reeve-Daly MP, Lander ES (1996) Parametric and nonparametric linkage analysis: a unified multipoint approach. Am J Hum Genet 58: 1347-1363.

10. Donnelly KP (1983) The probability that related individuals share some section of genome identical by descent. Theor Popul Biol 23: 34-63.

11. Abecasis GR, Cherny SS, Cookson WO, Cardon LR (2002) Merlin--rapid analysis of dense genetic maps using sparse gene flow trees. Nat Genet 30: 97-101.

12. Johnson AD, Handsaker RE, Pulit SL, Nizzari MM, O'Donnell CJ, et al. (2008) SNAP: a web-based tool for identification and annotation of proxy SNPs using HapMap. Bioinformatics 24: 2938-2939.

13. de Bakker PI, McVean G, Sabeti PC, Miretti MM, Green T, et al. (2006) A high-resolution HLA and SNP haplotype map for disease association studies in the extended human MHC. Nat Genet 38: 1166-1172.

14. Nelson MR, Bryc K, King KS, Indap A, Boyko AR, et al. (2008) The Population Reference Sample, POPRES: a resource for population, disease, and pharmacological genetics research. Am J Hum Genet 83: 347-358.

15. Purcell S, Neale B, Todd-Brown K, Thomas L, Ferreira MA, et al. (2007) PLINK: a tool set for whole-genome association and population-based linkage analyses. Am J Hum Genet 81: 559-575.

16. Price AL, Patterson NJ, Plenge RM, Weinblatt ME, Shadick NA, et al. (2006) Principal components analysis corrects for stratification in genome-wide association studies. Nat Genet 38: 904-909.

17. Ng PC, Henikoff S (2003) SIFT: Predicting amino acid changes that affect protein function. Nucleic Acids Res 31: 3812-3814.

18. Ng PC, Henikoff S (2006) Predicting the effects of amino acid substitutions on protein function. Annu Rev Genomics Hum Genet 7: 61-80.

19. Kumar P, Henikoff S, Ng PC (2009) Predicting the effects of coding non-synonymous variants on protein function using the SIFT algorithm. Nat Protoc 4: 1073-1081.

20. Ng PC, Henikoff S (2002) Accounting for human polymorphisms predicted to affect protein function. Genome Res 12: 436-446.

21. Adzhubei IA, Schmidt S, Peshkin L, Ramensky VE, Gerasimova A, et al. (2010) A method and server for predicting damaging missense mutations. Nat Methods 7: 248-249.

22. Ramensky V, Bork P, Sunyaev S (2002) Human non-synonymous SNPs: server and survey. Nucleic Acids Res 30: 3894-3900.

23. Sunyaev SR, Eisenhaber F, Rodchenkov IV, Eisenhaber B, Tumanyan VG, et al. (1999) PSIC: profile extraction from sequence alignments with position-specific counts of independent observations. Protein Eng 12: 387-394.

24. Kumar S, Suleski MP, Markov GJ, Lawrence S, Marco A, et al. (2009) Positional conservation and amino acids shape the correct diagnosis and population frequencies of benign and damaging personal amino acid mutations. Genome Res 19: 1562-1569.

25. Fitch W (1971) Toward Defining the Course of Evolution: Minimum Change for a Specific Tree Topology. Systematic Zoology 20: 406-416.

26. Kimura M (1968) Evolutionary rate at the molecular level. Nature 217: 624-626.

27. Vasen HF, Watson P, Mecklin JP, Lynch HT (1999) New clinical criteria for hereditary nonpolyposis colorectal cancer (HNPCC, Lynch syndrome) proposed by the International Collaborative group on HNPCC. Gastroenterology 116: 1453-1456.

28. Chamary JV, Parmley JL, Hurst LD (2006) Hearing silence: non-neutral evolution at synonymous sites in mammals. Nat Rev Genet 7: 98-108.

29. Kimchi-Sarfaty C, Oh JM, Kim IW, Sauna ZE, Calcagno AM, et al. (2007) A "silent" polymorphism in the MDR1 gene changes substrate specificity. Science 315: 525-528.

30. Yeo G, Burge CB (2004) Maximum entropy modeling of short sequence motifs with applications to RNA splicing signals. J Comput Biol 11: 377-394.

31. Rivas E, Eddy SR (2000) Secondary structure alone is generally not statistically significant for the detection of noncoding RNAs. Bioinformatics 16: 583-605.

32. Hofacker IL (2003) Vienna RNA secondary structure server. Nucleic Acids Res 31: 3429-3431.

33. Eyre-Walker AC (1991) An analysis of codon usage in mammals: selection or mutation bias? J Mol Evol 33: 442-449.

34. Ikemura T (1985) Codon usage and tRNA content in unicellular and multicellular organisms. Mol Biol Evol 2: 13-34.

35. Zhang G, Ignatova Z (2009) Generic algorithm to predict the speed of translational elongation: implications for protein biogenesis. PLoS One 4: e5036.

36. Ashley EA, Butte AJ, Wheeler MT, Chen R, Klein TE, et al. (2010) Clinical assessment incorporating a personal genome. Lancet 375: 1525-1535.

37. Bodenreider O (2004) The Unified Medical Language System (UMLS): integrating biomedical terminology. Nucleic Acids Res 32: D267-270.

38. Morgan AA, Chen R, Butte AJ (2010) Likelihood ratios for genome medicine. Genome Med 2: 30.
